# *OLMALINC*/OCT4/*BMP2* axis enhances osteogenic-like phenotype of renal interstitial fibroblasts to participate in Randall’s plaque formation

**DOI:** 10.1186/s10020-022-00576-4

**Published:** 2022-12-29

**Authors:** Zewu Zhu, Fang Huang, Yingcheng Jiang, Shuhao Ruan, Minghui Liu, Youjie Zhang, Yongchao Li, Jinbo Chen, Yu Cui, Zhiyong Chen, Hequn Chen, Feng Zeng

**Affiliations:** 1 Department of Urology, Xiangya Hospital, Central South University, Changsha, 410008 Hunan China; 2 Department of Internal Medicine, Section Endocrinology, Yale University School of Medicine, New Haven, CT USA

**Keywords:** Randall’s plaques, Renal interstitial fibroblasts, Osteogenic-like differentiation, *OLMALINC*, OCT4, *BMP2*

## Abstract

**Background:**

Randall’s plaques (RP) are identified as anchored sites for kidney calcium oxalate stones, but the mechanism remains unclear. Given the importance of osteogenic-like cells in RP formation and OCT4 in reprogramming differentiated cells to osteoblasts, the current study explored the potential role of OCT4 in RP formation.

**Methods:**

OCT4 and biomineralization were evaluated in RP, and immunofluorescence co-staining was performed to identify these cells with alteration of OCT4 and osteogenic markers. Based on the analysis of tissue, we further investigated the mechanism of OCT4 in regulating osteogenic-like differentiation of primary human renal interstitial fibroblasts (hRIFs) in vitro and vivo.

**Results:**

We identified the upregulated OCT4 in RP, with a positive correlation to osteogenic markers. Interestingly, fibroblast marker Vimentin was partially co-localized with upregulated OCT4 and osteogenic markers in RP. Further investigations revealed that OCT4 significantly enhanced the osteogenic-like phenotype of hRIFs in vitro and in vivo. Mechanically, OCT4 directly bound to *BMP2* promoter and facilitated its CpG island demethylation to transcriptionally promote *BMP2* expression. Furthermore, combination of RIP and RNA profiling uncovered that lncRNA *OLMALINC* physically interacted with OCT4 to promote its stabilization via disrupting the ubiquitination. Additionally, *OLMALINC* was upregulated in fibroblasts in RP visualized by FISH, and a positive correlation was revealed between *OLMALINC* and OCT4 in RP.

**Conclusions:**

The upregulation of OCT4 in hRIFs was a pathological feature of RP formation, and *OLMALINC*/OCT4/*BMP2* axis facilitated hRIFs to acquire osteogenic-like phenotype under osteogenic conditions, through which the pathway might participate in RP formation. Our findings opened up a new avenue to better understand RP formation in which osteogenic-like process was partially triggered by lncRNAs and pluripotency maintenance related genes.

**Supplementary Information:**

The online version contains supplementary material available at 10.1186/s10020-022-00576-4.

## Introduction

Nephrolithiasis afflicts about 6.4% population in China (Zeng et al. 2017) and 11% population in America (Hill et al. 2022). Kidney calcium oxalate (CaOx) stones are found in approximately 80% of patients (Khan et al. 2016), with a recurrence rate of 50% within 5–10 years (Eisner and Goldfarb 2014). Calcium phosphate (CaP) deposits at the tip of renal papillae, Randall’s plaques (RP) (Randall 1937; Evan et al. 2018), were identified to be an anchored site for CaOx stones (Miller et al. 2010; Williams et al. 2022). Interestingly, the structure of RP presents somewhat similarities to physiological bone formation as well as pathological biominerals driven by osteogenic-like cells (Khan and Gambaro 2007; Gay et al. 2020; Zhu et al. 2020a). Though significant progress has been achieved in the nanoscale analysis of RP over the last decades (Gay et al. 2020; Verrier et al. 2016; Evan et al. 2003a), and renal tubular epithelial cells, as well as renal interstitial fibroblasts, were reported to successfully form CaP crystal depositions (Priante et al. 2019; Zhu et al. 2021) under osteogenic induction, the mechanism underlying the very first step of CaP formation, one of the speculative ways in which some cells adopted osteogenic-like phenotype, remains poorly understood in RP formation.

In recent years, fibroblasts were reported to be directly converted into functional osteoblasts after transfection with defined factors (Yamamoto et al. 2015; Ahmed et al. 2019). The co-transfection of *Octamer binding transcription factor 4* (*OCT4*), *RUNX2*, *SP7,* and *L-Myc* reprogrammed human fibroblasts into osteoblast-like cells with a similar gene expression profile as well as osteogenic function to normal osteoblasts (Yamamoto et al. 2015). Additionally, co-transfection of *OCT4*, *C-Myc,* and lineage specific factor (*hLMP-3*) converted mouse embryonic fibroblast to osteoblast-like cells, transplantation of which yielded efficient healing in femoral bone defects induced in rat models (Ahmed et al. 2019). Thereinto, the critical role of OCT4 was highlighted during the conversion (Malik et al. 2018). OCT4, encoded by Pit-Oct-Unc (POU) domain class 5, transcription factor 1 (*POU5F1*), is well-established as one of the pluripotency-associated transcription factors (TF) maintaining embryonic stem cells state (Malik et al. 2018; Hurk et al. 2016), and OCT4 has been identified to be the only factor which cannot be substituted by other members of POU family (Jerabek et al. 2014). Meanwhile, previous studies have investigated the regulative role of OCT4 in osteogenic differentiation. *OCT4* knockdown markedly impaired the ability of mouse bone mesenchymal stem cells (BMSCs) to differentiate into osteoblasts (Malvicini et al. 2019); conversely, *OCT4* overexpression was reported to enhance osteogenic trans-differentiation of human umbilical vein endothelial cells induced with BMP4 (Kim et al. 2020). These studies were combined to indicate the critically promotive role of OCT4 in modulating osteogenic differentiation.

Intriguingly, the current study for the first time identified the elevation of OCT4 in RP and revealed the partial co-localization of upregulated OCT4 and fibroblast marker Vimentin in RP. Meanwhile, osteogenic markers were found to be upregulated in interstitial cells expressing Vimentin. Further study revealed that *OCT4* overexpression facilitated the osteogenic-like differentiation of human renal interstitial fibroblasts (hRIFs) in *vitro* and in *vivo*. Further investigations revealed that OCT4 bound to *BMP2* promoter as predicted by bioinformatic analysis and thus promoted *BMP2* transcription via decreasing its promoter CpG demethylation. Moreover, long noncoding RNA (lncRNA) *OLMALINC* stabilized OCT4 protein via directly binding to OCT4 to suppress its ubiquitylation. Our data uncovered the regulative role of *OLMALINC*/OCT4/*BMP2* axis in osteogenic-like differentiation of hRIFs, providing new insights into the mechanism of renal interstitium biomineralization, which serves as a stepping stone for understanding RP formation.

## Materials and methods

### Clinical samples

The study protocol was approved by Xiangya Hospital Ethics Committee (Proof Number: 201603035), and written informed consent were obtained from each patient. As described in our previous study (Zhu et al. 2020a), RP tissues were got from idiopathic CaOx stone formers (CaOx-SF) using biopsy forceps (Karl Storz, Germany) during percutaneous nephrolithotomy and normal renal papillae (NRP) tissues from patients with renal cancers receiving nephrectomy. The characteristics of included patients were summarized in Additional file 5: Table S1.

### Cell culture

As reported in our previous study (Zhu et al. 2020a), primary hRIFs were isolated from the normal renal medulla and identified by detecting Vimentin and E-cadherin, and hRIFs with passages in 3–6 were used for experiments. HRIFs were cultured in DMEM (BI, Israel) with 10% fetal bovine serum (BI, Israel) and 1% penicillin/streptomycin (BI, Israel), maintained at 37 °C with 5% CO_2_. Osteogenic-like differentiation of HRIFs was induced with osteogenic medium containing β-glycerophosphate (10 mM; Sigma, USA), ascorbic acid (200 μM; Sigma, USA) and dexamethasone (100 nM; Sigma, USA), as widely used in previous studies (Zhu et al. 2020a; Huang et al. 2015).

### Cell transfection

Re-constituted lentivirus purchased from GenePharma (Shanghai, China) were used to knockdown *OCT4* (Len-sh-*OCT4*), *OLMALINC* (Len-sh-*OLMALINC*) or *BMP2* (Len-sh-*BMP2*), and overexpress *OCT4* (Len-*OCT4*) or *OLMALINC* (Len-*OLMALINC*) in hRIFs, and the transfection of lentivirus carrying a scrambled sequence served as the negative control (Len-ctrl; Len-sh-ctrl). The designed sequences of shRNA were listed in Additional file 5: Table S2.

### Reverse transcription and quantitative real-time polymerase chain reaction (qRT-PCR)

Total RNA was isolated from hRIFs or tissues using SteadyPure Universal RNA Extraction Kit (AG, China). PrimeScript RT reagent Kit (Takara, Japan) was used to synthesize first-strand cDNAs from template RNA (1 ug), followed by qRT-PCR (QuantStudio5/7, USA) using SYBR Green PCR Kit (Takara, Japan). Relative gene expressions normalized to GAPDH were analyzed using 2^−ΔΔCt^ method. Primers designed for qPCR were listed in Additional file 5: Table S3.

### Sequencing for total RNA and RNA immunoprecipitation (RIP-sequencing)

HRIFs were cultured in normal medium or induced with osteogenic medium for a week, and total RNA was isolated for constructing cDNA libraries, followed by RNA sequencing based on Illumina platform (hiseq xten; RiboBio Co., Ltd., China). For RIP, as described in our previous study (Zhu et al. 2020a), Magna RIP Kit (17-701; Millipore, USA) was used to obtain those lncRNAs binding to OCT4 (ab184665, Abcam, UK), and normal mouse IgG was served as control. Total RNA isolated from OCT4 conjugated beads (n = 3) or input (n = 3) was sent for LncRNA sequencing (RiboBio Co., Ltd., China). To account for library depth as well as gene length, transcripts per kilobase of exon model per million mapped reads (TPM) was used to normalize read counts. Statistical analysis with DESeq2 was used to determine differentially expressed lncRNAs between normal group (n = 3) and osteogenic group (n = 3). Paired t-test with one side, reported in a previous study (Lenzen et al. xxxx), was used to analyze the significance of lncRNA enrichment in OCT4 Immunoprecipitation products versus the input, in which significance was identified as enrichment fold ≥ 4 and *P* ≤ 0.05.

### 5′ and 3′ rapid amplification of complementary DNA ends (5′ and 3′ RACE)

5′ and 3′ RACE were performed for *OLMALINC* using SMARTer RACE Kit (634858; Clontech, USA) following the manufacturer’s instructions. The products of 5’ and 3’ RACE was purified by 1% agarose gel electrophoresis, followed by ligation to vector and sequencing (Tsingke Biotechnology Co., Ltd., China). Additional file 5: Table S4 listed the gene-specific primers (GSPs) and nest gene-specific primers (NGSPs).

### Fluorescence in situ hybridization (FISH)

RNA-FISH for *OLMALINC* was performed with RNA Fluorescence in Situ Hybridization Kit (BersinBio, China) following the manufacturer’s protocols. Briefly, after being fixed and permeabilized, hRIFs were incubated with a Cy3-labeled specific probe for *OLMALINC* at 37 °C overnight. Next, hRIFs were stained with DAPI and imaged with confocal microscopy (Leica TCS SP8X, Germany). In terms of RNA-FISH for *OLMALINC* combined with immunofluorescence for Vimentin in renal papilla tissues, slides were prepared conventionally and co-staining was performed as described by a previous study (Liao et al. 2022).

### Immunoprecipitation (IP) and immunoblotting (IB)

For IP, anti-OCT4 antibody (ab184665, Abcam, UK) was incubated with protein A/G beads (Millipore, USA) at room temperature for 1 h. Next, the supernatant of cell lysis was incubated with beads conjugated with anti-OCT4 at 4 °C overnight, followed by elution with loading buffer at 98 °C for 5 min, and then subjected to IB. IB was performed in accordance with our previous study (Zhu et al. 2020a). The protein blots were visualized in ChemiDoc XRS (Bio-Rad, USA) using chemiluminescence (NCM Biotech; China). The details of primary antibodies and secondary antibodies were listed in Additional file 5: Table S5. The intensity of protein blots quantified by ImageJ software was normalized to that of GAPDH, which was expressed as the ratio of the control group.

### Chromatin immunoprecipitation (ChIP)

After shearing chromatin to fragments of about 200–1000 bp by a sonicator (Sonics and Materials, USA), OCT4-ChIP was performed using EpiQuik™ ChIP Kit (Epigentek, USA) in accordance with the manufacturer’s instructions. The immunoprecipitated products were analyzed by q-PCR, and the q-PCR products were verified by 1% agarose gel electrophoresis. Anti-RNA polymerase II (RPII; involved in the kit) and primers for regions of *GAPDH* promoter were served as a positive control (PC); normal mouse IgG (involved in the kit) as a negative control. Additional file 5: Table S6 listed the primers designed for amplifying regions of *BMP2* or *SMAD4* promoter containing putative OCT4 binding sites.

### Bisulfite sequencing PCR (BSP)

Genomic DNA was extracted and bisulfite converted using Methyl Detector TM Bisulfite Modification Kit (Active Motif, USA). The CpG islands of *BMP2* promoter were predicted by website (Li and Dahiya 2002), followed by amplifying with PCR using designed primers flanking these regions (Additional file 5: Table S7). Ligated pCR2.1-TOPO vector (Invitrogen, USA) carrying PCR products was transfected into E. cloni. After selection with ampicillin, 5 clones were randomly picked for sequencing. BISMA software was used to analyze CpG methylation.

### Luciferase reporter assay

Luciferase reporter assay was performed using the Dual‐Luciferase Reporter assay system (Promega, USA). Briefly, the pGL3-basic luciferase reporter vector (Promega, USA) was constructed with a *BMP2* promoter region containing putative OCT4 binding sites or the corresponding mutant sites. The luciferase reporter vector was co-transfected with *OCT4* plasmid (p-*OCT4*) in hRIFs with Lipo2000 (Invitrogen, USA) for 48 h, and the dual-luciferase activity (firefly luciferase/renilla luciferase) was determined.

### Alkaline phosphatase (ALP) activity assay and alizarin red staining (ARS)

ALP activity of hRIF lysis was determined with an ALP colorimetric assay kit (Beyotime, China), and calcium deposits in hRIF layers was stained with Alizarin Red (ARS; PH = 4.0, Solarbio, China), as described in our previous study (Zhu et al. 2020a).

### Immunohistochemistry (IHC), immunofluorescence, Von Kossa and Masson's trichrome staining

IHC for RP and NRP tissues was performed in accordance to our previous study (Zhu et al. 2020a). Regarding immunofluorescence of frozen tissue sections, briefly, fresh tissues were immediately snap frozen and embedded with OCT compound (SAKURA, Japan). 8 µm tissue slices were prepared (Leica CM1950, Germany), followed by adding Fixation Buffer (− 20 °C) for 15 min. After blocking with 10% goat serum at room temperature (Solarbio, China) for 60 min, slices were incubated with primary antibodies (anti-Vimentin, 1:300, #5741, CST, USA/anti-Vimentin, 1:300, ab8069, Abcam, UK; anti-OCT4, 1:300, ab184665, Abcam, UK; anti-RUNX2, 1:500, #12556, CST, USA; anti-OCN, 1:400, 23418-1-AP, Proteintech, China) at 4 °C overnight. After incubation with Alexa Fluorophore 488 (1:600, ab150077/ab150113, Abcam, UK) or 647 (1:600, ab150115/ab150083, Abcam, UK) conjugated antibodies at room temperature for 60 min, followed by DAPI (Service, China) staining for 5 min, images were visualized using a confocal microscope system (Leica TCS SP8 X, Germany). Von-Kossa Kit (Solaribo, China) was used to stain calcium deposits in renal papillae according to the manufacturer’s instructions, and then was counterstained with hematoxylin and eosin (HE). HE (Solaribo, China) and Masson's trichrome staining (Solaribo, China) were performed using ready-to-use kit according to the manufacture’s instructions.

As described by our previous study (Zhu et al. 2021), semi-quantitative analysis was performed for IHC staining and the co-localization ratio in immunofluorescence. Briefly, IHC staining was analyzed using the “IHC Toolbox” plugin in Image J (Shu et al. 2016), and the co-localization ratio was calculated by the confocal microscope system (Leica TCS SP8 X, Germany). Semi-quantitative analysis of Masson's trichrome staining was performed using “color deconvolution” plugin in Image J (Ruifrok and Johnston 2001), as described by previous studies (Sarila et al. 2020; Pazos et al. 2010). The blue-stained collagen fibers were measured as the collagen volume fraction (CVF) of the region of interest. All the stained pictures were analyzed with blind.

### Subcutaneous ectopic implantation of osteogenic-induced hRIFs in vivo

This study obtained the approval from the Institutional Experimental Animal Committee of Central South University (Proof Number: 2021101006), and we made all efforts to reduce number and the suffering of mice. A total of 21 eight-week-old female nude mice were used, which were provided by the Animal Experimental Center of Central South University. All hRIFs with or without lentiviral transduction were osteogenic induced for 14 days, and then 5 × 10^6^ cells of each group were loaded onto 25 mg of porous bone mineral substitute granules (Geistlich Bio-Oss, Switzerland) mixed with 200ul DMEM containing 10% fetal bovine serum (BI, Israel) and 1% penicillin/streptomycin (BI, Israel). After incubation at 37 °C for 1 h, implants were subcutaneously placed on 2 different sites in the dorsum of each mouse under general anesthesia, as described in a previous study (Pillai et al. 2017). To evaluate whether hRIFs survived, hRIFs transfected with lentivirus carrying sequence encoding luciferase were osteogenic induced for 14 days, followed by implantation in 3 mice. 8 weeks after subcutaneous implantations, the fluorescence was detected using animal fluorescence imaging (IVIS Spectrum, PerkinElmer, USA) in 15 min after d-Luciferin (YEASEN, China) injection. To determine the role of OCT4 in osteogenic-like phenotype of hRIFs in vivo, we designed 6 groups including the blank group (granules without cells), the normal control (NC) group (granules with hRIFs), the Len-ctrl group (granules with hRIFs transfected with Len-ctrl) and the Len-*OCT4* group (granules with hRIFs transfected with Len-*OCT4*), Len-sh-ctrl group (granules with hRIFs transfected with Len-sh-ctrl) and the Len-sh-*OCT4* group (granules with hRIFs transfected with Len-sh-OCT4), and each group was implanted in 6 subcutaneous sites in different mice. Mice (N = 18) were euthanized and implants were collected 8 weeks after subcutaneous implantations. Samples were fixed by 4% paraformaldehyde for 48 h, and then were decalcified with EDTA for 3 weeks prior to perform HE, Masson's trichrome, and IHC staining.

### Statistical analysis

All experiments were repeated independently with similar results at least three times. Categorical variables were compared by Chi-squared test or Fisher’s exact test; quantitative data expressed as mean ± SD were compared by one-way ANOVA or unpaired Student’s *t*-test. Correlations between two parameters were determined with Spearman’s rank correlation. A value of two-tailed *P* ≤ 0.05 was considered statistically significant. All statistical analysis was carried out using GraphPad Prism 8 software (GraphPad Software, USA). In all comparisons, * was defined as *P* < 0.05, ** as *P* < 0.01, and *** as *P* < 0.001.

## Results

### OCT4 was upregulated in RP and osteogenic induced hRIFs

**Fig. 1:**
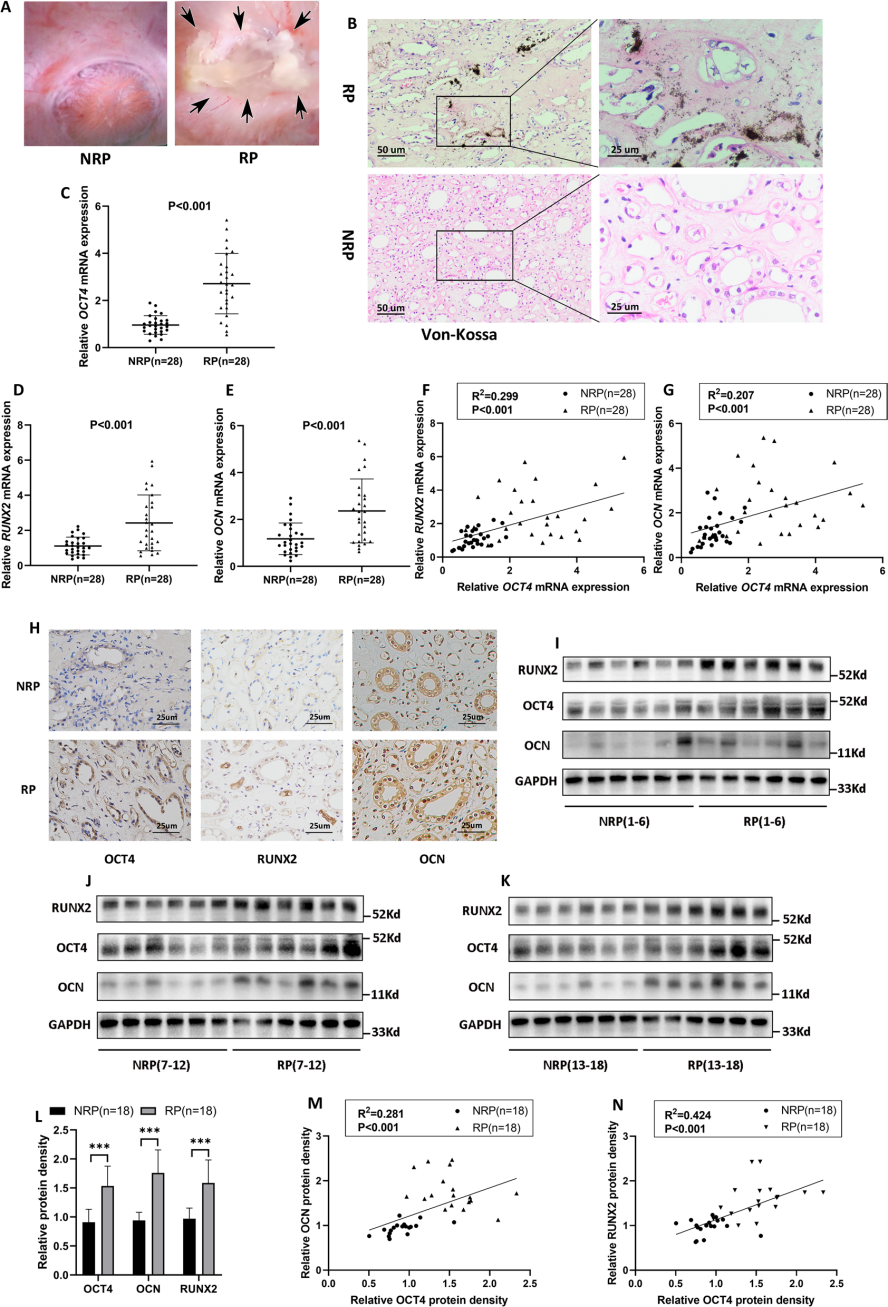
OCT4 and osteogenic related markers were upregulated in Randall’s plaques (RP).** A** Endoscopic images of normal renal papillae (NRP) and RP attached with a renal stone (black arrows). **B** Representative images of Von Kossa staining for calcium deposits (black granules) in NRP. **C–E** QRT-PCR analysis of *OCT4*, *RUNX2*, *OCN* mRNA expression in RP (n = 28) and NRP (n = 28). **F, G** Liner regression analysis of *OCT4* mRNA expression and *RUNX2* as well as *OCN* mRNA expression in RP (n = 28) and NRP (n = 28). **H** Representative images of IHC for OCT4, RUNX2, OCN in RP (N = 8) and NRP (N = 8). **I–L** Immunoblotting (IB) analysis of OCT4, RUNX2 and OCN in RP (n = 18) and NRP (n = 18). **M, N** Liner regression analysis of OCT4 protein density and RUNX2 as well as OCN protein density in RP (n = 18) and NRP (n = 18)

**Fig. 2:**
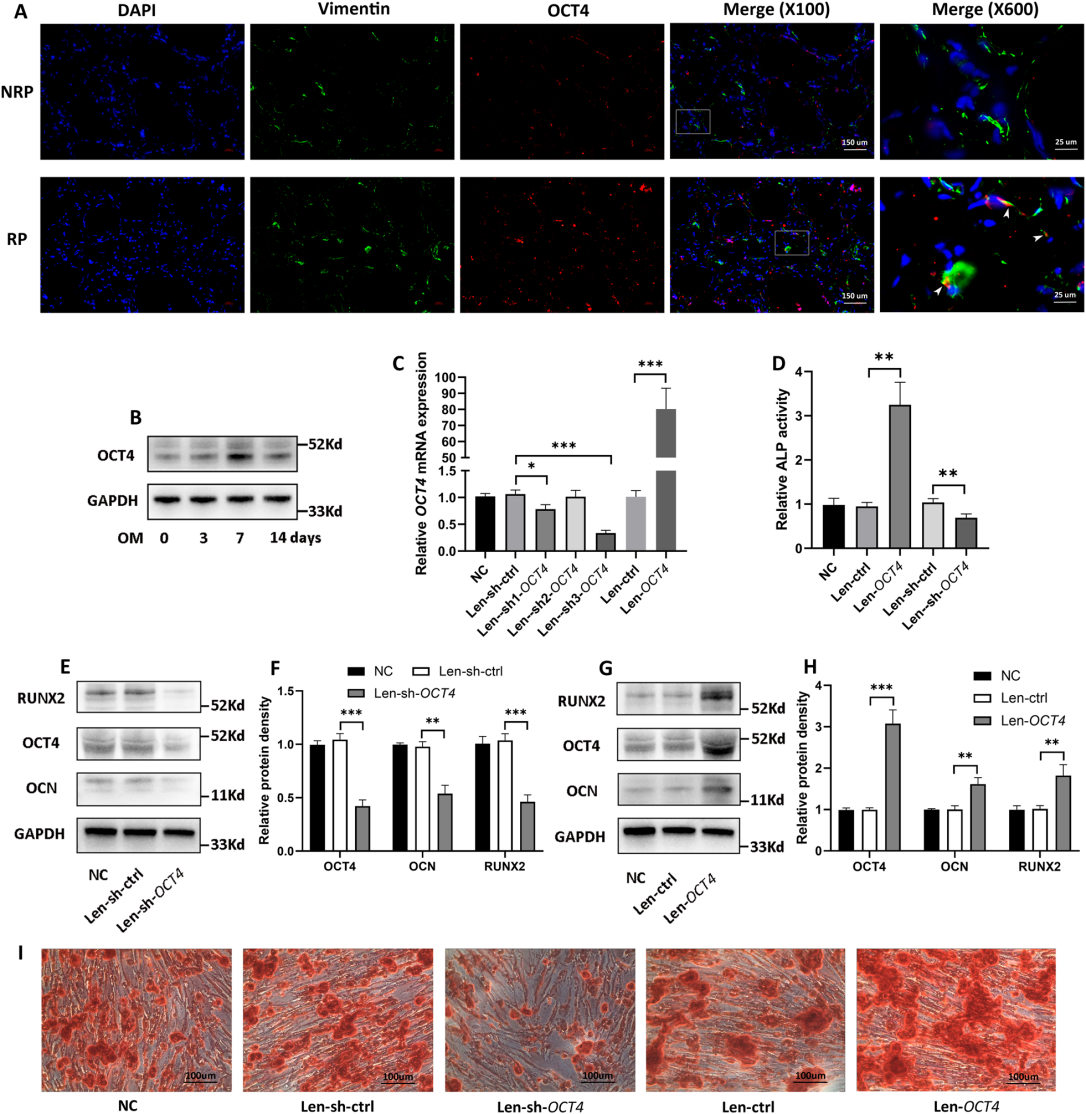
OCT4 enhanced osteogenic-like differentiation of human renal interstitial fibroblasts (hRIFs) in vitro. **A** Representative immunofluorescence co-staining images of Vimentin (Green) and OCT4 (Red) in Randall’s plaques (RP; n = 6) and normal renal papillae (NRP; n = 6). **B** Immunoblotting (IB) analysis of OCT4 in hRIFs 0, 3, 7, and 14 days after osteogenic medium (OM) induction (n = 3). **C** HRIFs were transfected with recombinant lentivirus to overexpress or silence *OCT4*, and qRT-PCR determined the efficiency (n = 3). Len-sh3-*OCT4* was selected for silencing *OCT4* in the following experiments because of the highest efficiency. **D** Alkaline phosphatase (ALP) activity in cell lysis of transfected hRIFs 7 days after osteogenic induction (n = 3). **E, F** IB analysis of OCT4, RUNX2, and OCN in *OCT4* silenced hRIFs 7 days after osteogenic induction (n = 3). **G, H** IB analysis of OCT4, RUNX2, and OCN in *OCT4* overexpressed hRIFs 7 days after osteogenic induction (n = 3). **I** Alizarin Red Staining (ARS) for calcium nodes in transfected hRIFs 14 days after osteogenic induction (n = 3)

As shown in Von Kossa staining, extensive calcium deposits were distributed in the interstitium of RP (Fig. 1A, B), whereas it was negative in NRP (Fig. 1A, B). To evaluate whether OCT4 participated in the process of renal interstitial calcification proceeding RP formation, we detected both mRNA and protein expression of *OCT4* in RP and NRP tissues, and these results consistently showed the upregulation of OCT4 in RP (Fig. 1C, H–L; Additional file 1: Fig. S1A). Moreover, *OCT4* expression demonstrated a positive correlation with that of the osteogenic-related genes (*OCN*, *RUNX2*) in both mRNA (Fig. 1D–G) and protein levels (Fig. 1M, N). Considering that incipient calcium deposits partially appeared in renal interstitium which was far apart from these cells with a tubular structure (Fig. 1B); renal interstitial calcium deposits were reported to grow by the addition of crystals on the periphery within a collagen framework (Khan et al. 2012), and the osteogenic capability of hRIFs was significantly stronger than that of tubular epithelial cells as revealed by our previous study (Zhu et al. 2021), we wondered whether OCT4 and osteogenic markers were altered in renal interstitial fibroblasts. Intriguingly, immunofluorescence co-staining showed that fibroblast marker Vimentin was partially co-localized with upregulated OCT4 (Fig. 2A, white arrow; Additional file 1: Fig. S1D) and osteogenic markers (Additional file 1: Fig. S1B–D, white arrow) in RP. Therefore, we induced osteogenic-like differentiation of hRIFs as in our previous study (Zhu et al. 2021), and OCT4 was also found to be significantly elevated in a time-dependent manner during osteogenic induction (Fig. 2B; Additional file 2: Fig. S2A). Based on these data, OCT4 and osteogenic-like fibroblasts were implicated to participate in RP formation.

### OCT4 enhanced osteogenic-like differentiation of hRIFs in *vitro* and in *vivo*

We proceeded to determine if OCT4 regulated osteogenic-like differentiation of hRIFs through ectopic overexpression and knockdown of *OCT4* with recombinant lentivirus (Fig. 2C). *OCT4* overexpression significantly promoted ALP activity (Fig. 2D) as well as the protein expression of osteogenic-related genes (Fig. 2E–H) in hRIFs cultured with osteogenic medium for 7 days, and also increased the calcium deposits detected by ARS in hRIFs cultured with osteogenic medium for 14 days (Fig. 2I). Inversely, *OCT4* knockdown markedly suppressed the osteogenic-like phenotype (Fig. 2D–I).

**Fig. 3:**
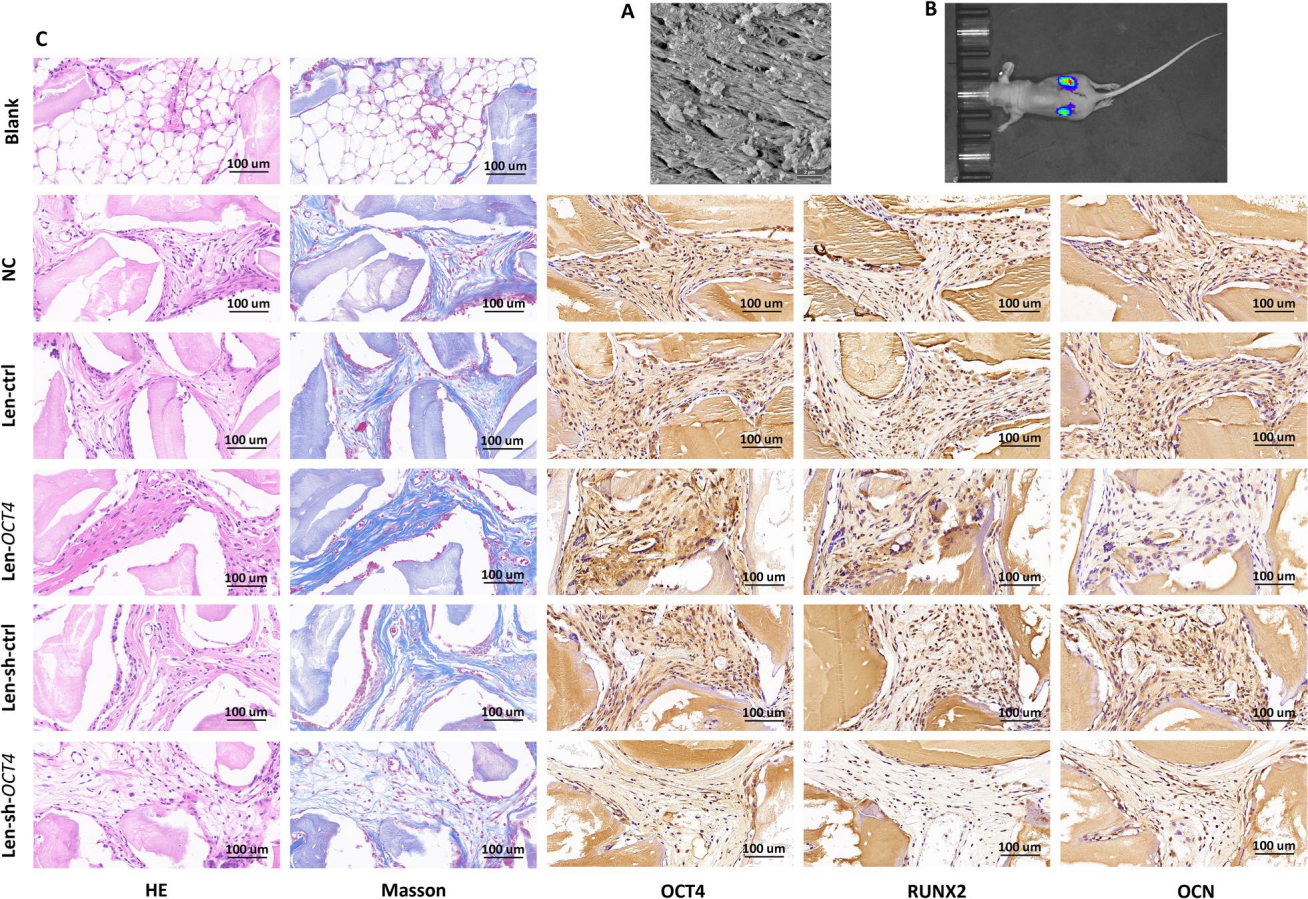
OCT4 enhanced osteogenic-like differentiation of human renal interstitial fibroblasts (hRIFs) in vivo.** A** The porous structure of bone mineral substitute granules visualized by scanning electron microscopy (X20000). **B** A representative fluorescence image of a nude female mouse 8 weeks after subcutaneous implantations carrying transfected hRIFs expressing luciferase (n = 3). **C** HE and Masson staining of serial sections from the blank group (granules without cells), the normal control (NC) group (granules with hRIFs), the Len-ctrl group (granules with hRIFs transfected with Len-ctrl), and the Len-*OCT4* group (granules with hRIFs transfected with Len-*OCT4*), Len-sh-ctrl group (granules with hRIFs transfected with Len-sh-ctrl) and the Len-sh-*OCT4* group (granules with hRIFs transfected with Len-sh-OCT4). IHC (OCT4; RUNX2; OCN) staining of serial sections from the NC group, the Len-ctrl group and the Len-*OCT4* group, Len-sh-ctrl group, and the Len-sh-*OCT4* group. Each of the 6 groups was implanted in 1 subcutaneous site of 6 mice and collected 8 weeks after implantation

We further conducted nude mouse subcutaneous ectopic implantation of porous bone mineral substitute granules (Fig. 3A) carrying osteogenic-induced hRIFs to determine the regulatory role of OCT4 in *vivo*. Above all, transfected hRIFs expressing luciferase were implanted to track whether hRIFs survived, and fluorescence imaging of the nude mouse dorsal implants showed that hRIFs survived 8 weeks after subcutaneous implantations (Fig. 3B), which was echoed to results that the blank group yielded empty bubble-like structures, while other implants carrying hRIFs showed ordered collagen fibers (Fig. 3C). Moreover, HE and Masson’s trichrome staining illustrated that OCT4 overexpression significantly enriched collagen fibers, while OCT4 knockdown significantly decreased collagen fibers (Fig. 3C; Additional file 2: Fig. S2B). Additionally, as revealed by IHC staining, OCT4 overexpression promoted RUNX2 but not OCN (Fig. 3C; Additional file 2: Fig. S2C), while OCT4 knockdown significantly suppressed both RUNX2 and OCN (Fig. 3C; Additional file 2: Fig. S2C). Taken together, these results verified that OCT4 functioned as a master regulator in promoting osteogenic-like differentiation of hRIFs both in *vitro* and in *vivo*.

### OCT4 directly bound to *BMP2* promoter and decreased its DNA methylation

**Fig. 4:**
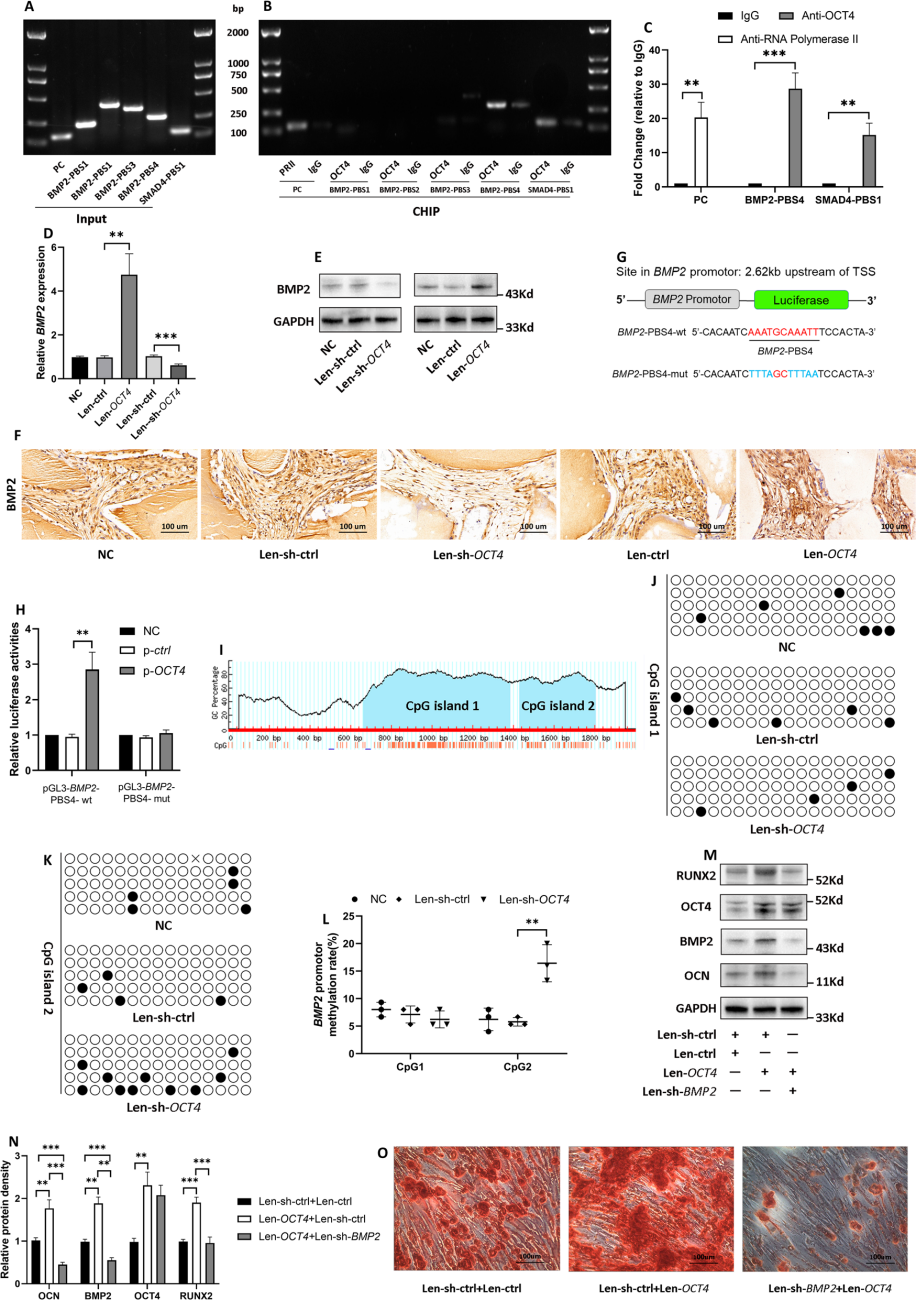
OCT4 directly bound to *BMP2* promoter and decreased its CpG methylation.** A, B** Agarose gel electrophoresis showed the PCR products of input and OCT4-ChIP; positive control (PC) was performed by amplifying the *GAPDH* promoter in RNA polymerase II (RPII)-ChIP; normal mouse IgG as a negative control. *BMP2*-PBS1/2/3/4 = *BMP2* promoter binding site 1/2/3/4; *SMAD4*-PBS1 = *SMAD4* promoter binding site 1. **C** Q-PCR was used to analyze the result of ChIP (n = 3), and the data were expressed as the fold change to the negative control. **D–E** QRT-PCR and immunoblotting (IB) analysis of BMP2 expression of human renal interstitial fibroblasts (hRIFs) transfected with Len-*OCT4* or Len-sh-*OCT4* (n = 3). **F** IHC staining for BMP2 of the normal control (NC) group (granules with hRIFs), the Len-ctrl group (granules with hRIFs transfected with Len-ctrl), and the Len-*OCT4* group (granules with hRIFs transfected with Len-*OCT4*), Len-sh-ctrl group (granules with hRIFs transfected with Len-sh-ctrl) and the Len-sh-*OCT4* group (granules with hRIFs transfected with Len-sh-OCT4); n = 6. **G** A schematic diagram illustrating the pGL3-basic luciferase reporter vector carrying the predicted sequence of *BMP2*-PBS4 (*BMP2*-PBS4-wt) or the mutant sequence (*BMP2*-PBS4-mut). **H** P-ctrl or p-*OCT4* and pGL3-*BMP2*-PBS4-wt or pGL3-*BMP2*-PBS4-mut were co-transfected to hRIFs, and the relative luciferase activity (firefly/renilla) was determined (n = 3). **I** Two predicted CpG islands in *BMP2* promoter. **J–L** Len-sh-*OCT4* or Len-sh-ctrl transfected hRIFs were induced with osteogenic medium for 7 days, and bisulfite sequencing PCR (BSP) was used to analyze the CpG methylation in predicted islands (n = 3). Black circle = methylation site; white circle = unmethylation site. **M, N** HRIFs were co-transfected with either Len-ctrl or len-*OCT4* in conjunction with Len-sh-ctrl or Len-sh-*BMP2*, and IB determined the protein expression of OCT4, OCN, BMP2, and RUNX2 7 days after osteogenic induction (n = 3); **O** Alizarin Red Staining (ARS) for calcium nodes 14 days after osteogenic induction (n = 3)

Considering that OCT4 has emerged as a transcription factor to regulate a set of genes via especially recognizing and binding to DNA regulatory regions (Malik et al. 2018), we used FIMO software (Grant et al. 2011) based on TF-motifs (Bailey et al. 2009), JASPAR dataset (Fornes et al. 2020) and HOCOMOCO dataset (Kulakovskiy et al. 2018) to predict whether OCT4 bound to the promoter of genes with a critical role in the early stage of osteogenic differentiation, including *BMP2* (Kostina et al. 2021), *SMAD2 *(Afzal et al. 2005), *SMAD4 *(Park et al. 2019), *RUNX2* (Kostina et al. 2021), *SP7* (Fu et al. 2018), *MSX2* (Satokata et al. 2000). Interestingly, OCT4 was predicted to bind to 4 sites of *BMP2* promoter and 1 site of *SMAD4* promoter (Additional file 5: Table S8), and ChIP-qPCR assay (Fig. 4A–C) showed that OCT4 directly bound to the site in *BMP2* promoter region located in 2.62 kb upstream of transcriptional start site (TSS; *BMP2*-PBS4), and bound to the site in *SMAD4* promoter region located in 2.01 kb upstream of TSS (*SMAD4*-PBS1). Given that *BMP2*-PBS4 had a higher fold change to IgG group than that of *SMAD4*-PBS1 (28.7 ± 4.7 vs 15.2 ± 3.4; P = 0.016; Fig. 4C), we further performed luciferase reporter assay to explore whether OCT4 could activate the transcription of *BMP2* via binding to its promoter as indicated by ChIP. Co-transfection of p-*OCT4* significantly augmented the relative luciferase activity in hRIFs with pGL3-basic vector carrying the wild type of *BMP2*-PBS4, while the mutation of *BMP2*-PBS4 substantially abolished the effect (Fig. 4G, H). Meanwhile, overexpression of *OCT4* significantly promoted both mRNA and protein expression of *BMP2*, and knockdown of *OCT4* showed the opposite effect (Fig. 4D, E; Additional file 2: Fig. S2D, E). We further verified the promotive role of *OCT4* in BMP2 in *vivo* by performing IHC staining for BMP2 in implants carrying hRIFs with overexpression or knockdown of *OCT4* (Fig. 4F; Additional file 2: Fig. S2F).

The methylation of regulatory regions can be altered when transcription factors are bound (Héberlé and Bardet 2019; Kurotaki et al. 2020), leading us to speculate whether OCT4 decreased the methylation of *BMP2* promoter to epigenetically promote its transcription. BSP was used to determine the methylation level of 2 predicted CpG islands in *BMP2* promoter (Fig. 4I), which showed that *OCT4* knockdown significantly increased the methylation of CpG island 2 (Fig. 4K, L; Additional file 3: Fig. S3) but not CpG island 1 (Fig. 4J, L; Additional file 4: Fig. S4) in osteogenic induced hRIFs. Moreover, *BMP2* silence with recombinant lentivirus was found to abolish the promotive effect of OCT4 on osteogenic-like differentiation of hRIFs (Fig. 4M, O; Additional file 2: Fig. S2G, H). The above results suggested that OCT4 decreased the DNA methylation of *BMP2* promoter to enhance its transcription, partially through which OCT4 promoted osteogenic-like differentiation of hRIFs.

### lncRNA *OLMALINC* functioned as an upstream regulator of OCT4

**Fig. 5:**
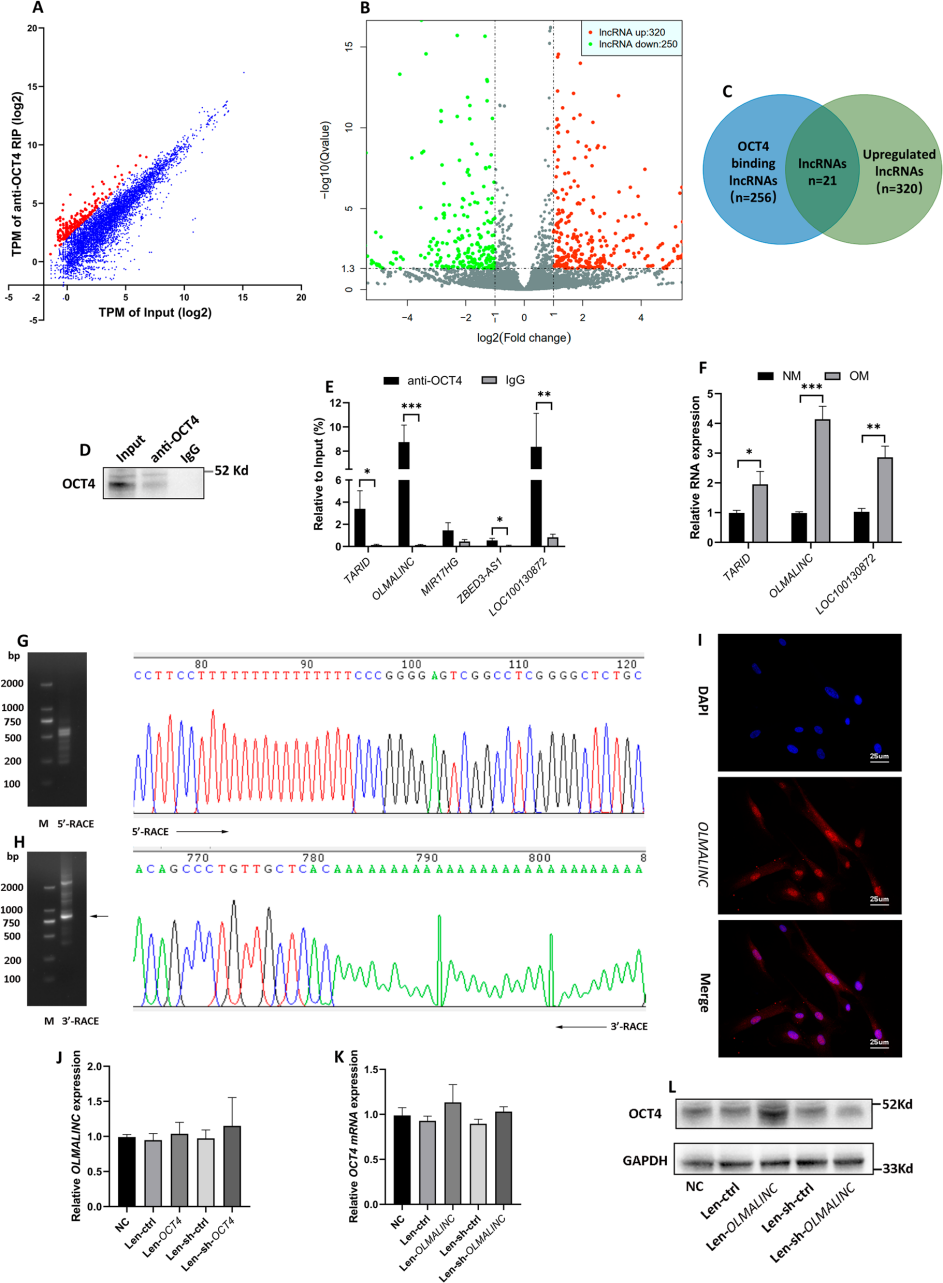
*OLMALINC*, an upregulated lncRNA in osteogenic medium induced human renal interstitial fibroblasts (hRIFs), directly bound to OCT4 and elevated OCT4 protein level.** A** OCT4-RIP-Seq was performed in hRIFs induced with osteogenic medium for a week. Data were expressed as mean adjusted TPM (log2); n = 3. Red dots showed the transcripts of lncRNAs with enrichment in OCT4-RIP more than fourfold to input. **B** RNA sequencing for total RNA was performed to identify differentially expressed lncRNAs between osteogenic group (7 days; n = 3) and normal group (7 days; n = 3). Red dots showed the transcripts of lncRNAs with fold change > 2 and Q value < 0.05. **C** Venn diagram illustrated the intersection of OCT4 binding lncRNAs identified by RIP-Seq and upregulated lncRNAs identified by RNA profiling. **D–E** RIP-qPCR was performed to verify those OCT4 binding lnRNAs with top 5 enrichment fold among the intersection of OCT4-RIP-Seq and RNA sequencing for total RNA; n = 3. **F** QRT-PCR analysis of the expression of *TARID*, *OLMALINC,* and *LOC100130872* in hRIFs cultured in either osteogenic medium (n = 3) or normal medium(n = 3) for 7 days. **G, H** Agarose gel electrophoresis showed the PCR products of 5′ and 3′ RACE for *OLMALINC*, and the sequences of 5′ and 3′ tail end were shown. **I** The location of *OLMALINC* determined by fluorescence in situ hybridization (FISH); n = 3. **J** QRT-PCR analysis of *OLMALINC* in hRIFs with overexpressed or silenced *OCT4*; n = 3. **K, L** QRT-PCR and immunoblotting (IB) analysis of OCT4 in hRIFs with overexpressed or silenced *OLMALINC*; n = 3

LncRNAs have emerged as critical regulators in cell differentiation (Ju et al. 2019), and interaction with RNA-binding proteins (RBPs) is one of an essential ways to function as regulators (Noh et al. 2018). Therefore, we proceeded to focus on those lncRNAs directly interacting with OCT4 protein through which lncRNAs might regulate OCT4 expression. OCT4-RIP-Seq was performed in hRIFs induced with osteogenic medium for a week, and 256 LncRNAs were identified to be enriched in OCT4 immunoprecipitation (Fig. 5A). Meanwhile, RNA sequencing for total RNA was performed to identify differentially expressed lncRNAs between normal and osteogenic group, and 320 LncRNAs were identified to be significantly upregulated in osteogenic group (Fig. 5B). We further focused on the intersection (Fig. 5C; Additional file 5: Table S9) of OCT4 binding lncRNAs and osteogenic induced upregulated lncRNAs, and RIP-qPCR was performed to verify those OCT4 binding lnRNAs with top 5 enrichment folds among the intersection (Fig. 5D, E). The results showed that *TARID*, *OLMALINC,* and *LOC100130872* were significantly enriched in OCT4 immunoprecipitation relative to IgG (Fig. 5D, E), and all of them were found to be markedly upregulated in osteogenic induced hRIFs (Fig. 5F). Thereinto, *OLMALINC* caught our attention due to the highest fold change in both OCT4 immunoprecipitation and osteogenic induced hRIFs among 3 identified lncRNAs.

We performed 5′ and 3′ RACE to determine the transcription of *OLMALINC* in hRIFs, and the results showed that the transcript sequence of *OLMALINC* was 1023 bp (Fig. 5G, H; Additional file 5: Table S10), which was slightly longer than that of the transcript variant (NR_026762.1; 103 bp) deposited in the NCBI database (Additional file 5: Table S10). FISH further showed that *OLMALINC* was located in both nuclear and cytoplasm (Fig. 5I). To determine the potential regulatory relationship between OCT4 and *OLMALINC*, the RNA interference of OCT4 or *OLMALINC* was performed using the recombined lentivirus, respectively. The expression of *OLMALINC* was not altered by neither OCT4 overexpression nor knockdown (Fig. 5J), while OCT4 protein expression was promoted by *OLMALINC* overexpression and decreased by *OLMALINC* knockdown (Fig. 5L; Additional file 2: Fig. 2I, J), despite of no change in the level of mRNA expression (Fig. 5K). The above findings indicated that *OLMALINC* participated in regulating the synthesis and/or degradation of OCT4 protein.

### *OLMALINC* elevated OCT4 protein level via inhibiting its ubiquitination

**Fig. 6:**
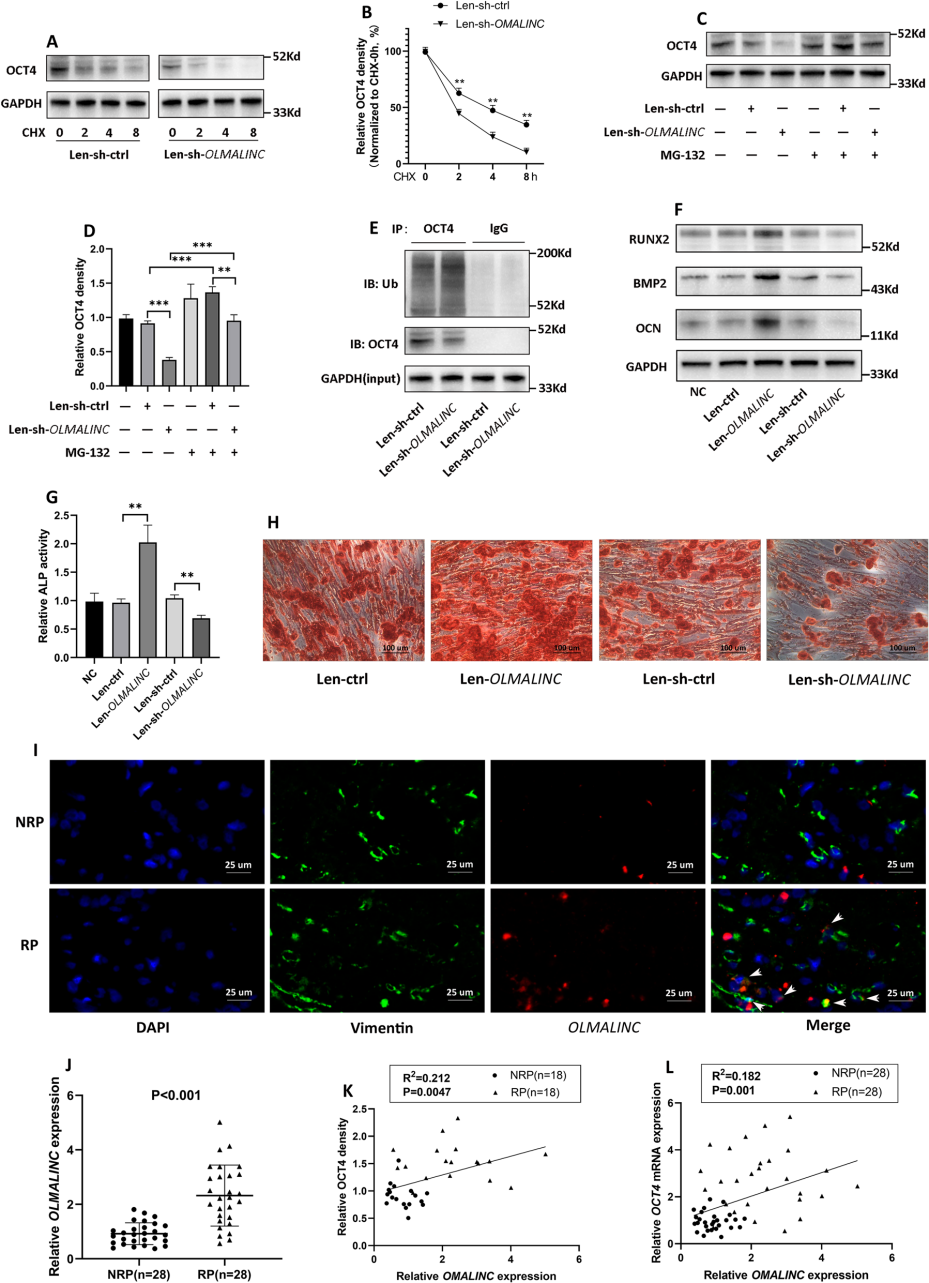
*OLMALINC* elevated OCT4 protein level via inhibiting its ubiquitination.** A, B** Len-sh-ctrl or Len-sh-*OLMALINC* transfected human renal interstitial fibroblasts (hRIFs) were pretreated with CHX (10 μM), and OCT4 was determined by Immunoblotting (IB) at the indicated time points (n = 3). **C, D** HRIFs transfected with Len-sh-ctrl or Len-sh-*OLMALINC* were treated with the proteasome inhibitor MG-132 (10um/ml) for 8 h or not, and OCT4 was determined by IB (n = 3). **E** HRIFs transfected with Len-sh-ctrl or Len-sh-*OLMALINC* were treated with MG-132 for 8 h, and the cell lysis was collected for immunoprecipitation (IP; n = 4). The immune-precipitates of anti-OCT4 or IgG (negative control) were incubated with anti-ubiquitin. **F** IB analysis of OCN, BMP2 and RUNX2 in hRIFs with overexpressed or silenced *OLMALINC* 7 days after osteogenic induction (n = 3). **G** Alkaline phosphatase (ALP) activity of hRIFs with overexpressed or silenced *OLMALINC* 7 days after osteogenic induction (n = 3). **H** Alizarin Red Staining (ARS) for calcium nodes in hRIFs with overexpressed or silenced *OLMALINC* 14 days after osteogenic induction (n = 3). **I** Representative co-staining images of RNA FISH for *OLMALINC* (Red) and immunofluorescence for Vimentin (Green) in Randall’s Plaques (RP; n = 6) and normal renal papillae (NRP; n = 6). **J** QRT-PCR analysis of *OLMALINC* expression in RP (n = 28) and NRP (n = 28). **K** Liner regression analysis of *OLMALINC* expression and OCT4 protein density in RP (n = 18) and NRP (n = 18). **L** Linear regression analysis of *OLMALINC* expression and *OCT4* mRNA expression in RP (n = 28) and NRP (n = 28)

To determine whether *OLMALINC* mediated the degradation of OCT4 protein, hRIFs were treated with the protein synthesis inhibitor cycloheximide (CHX). Interestingly, knockdown of *OLMALINC* markedly shortened the half-life of OCT4 (Fig. 6A, B). Since the ubiquitin–proteasome system has been well-established to play a vital role in mediating the protein stability and our study verified the direct interaction of *OLMALINC* and OCT4 protein, we speculated that *OLMALINC* might influence the degradation of OCT4 via disrupting the ubiquitin‐proteasome system. HRIFs transfected with Len-sh-*OLMALINC* were treated with the proteasome inhibitor MG-132 for 8 h, which dramatically recovered the OCT4 protein level suppressed by *OLMALINC* knockdown (Fig. 6C, D). Furthermore, *OLMALINC* knockdown was found to significantly enhance the ubiquitination level of OCT4 (Fig. 6E; Additional file 2: Fig. S2K).

### *OLMALINC*/OCT4/*BMP2* axis promoted osteogenic-like differentiation of hRIFs

Since *OLMALINC* stabilized OCT4 which decreased the methylation of *BMP2* promoter, we further explored the effect of *OLMALINC* on osteogenic-like differentiation of hRIFs. As expected, *OLMALINC* overexpression enhanced the protein level of osteogenic-related genes as well as ALP activities (Fig. 6F, G; Additional file 2: Fig. S2L), and *OLMALINC* knockdown yielded the opposite results (Fig. 6F, G; Additional file 2: Fig. S2L). Additionally, the promotive role of OLMALINC in osteogenic-like differentiation of hRIFs was verified by analyzing calcium deposits with ARS (Fig. 6H).

Moreover, the FISH result showed that *OLMALINC* was upregulated in fibroblasts visualized by Vimentin immunofluorescence in RP (Fig. 6I; Additional file 2: Fig. S2M), and q-PCR verified the significant upregulation of *OLMALINC* in RP (Fig. 6J), showing a positive correlation with *OCT4* in both protein (Fig. 6K) and mRNA (Fig. 6L) levels.

**Fig. 7:**
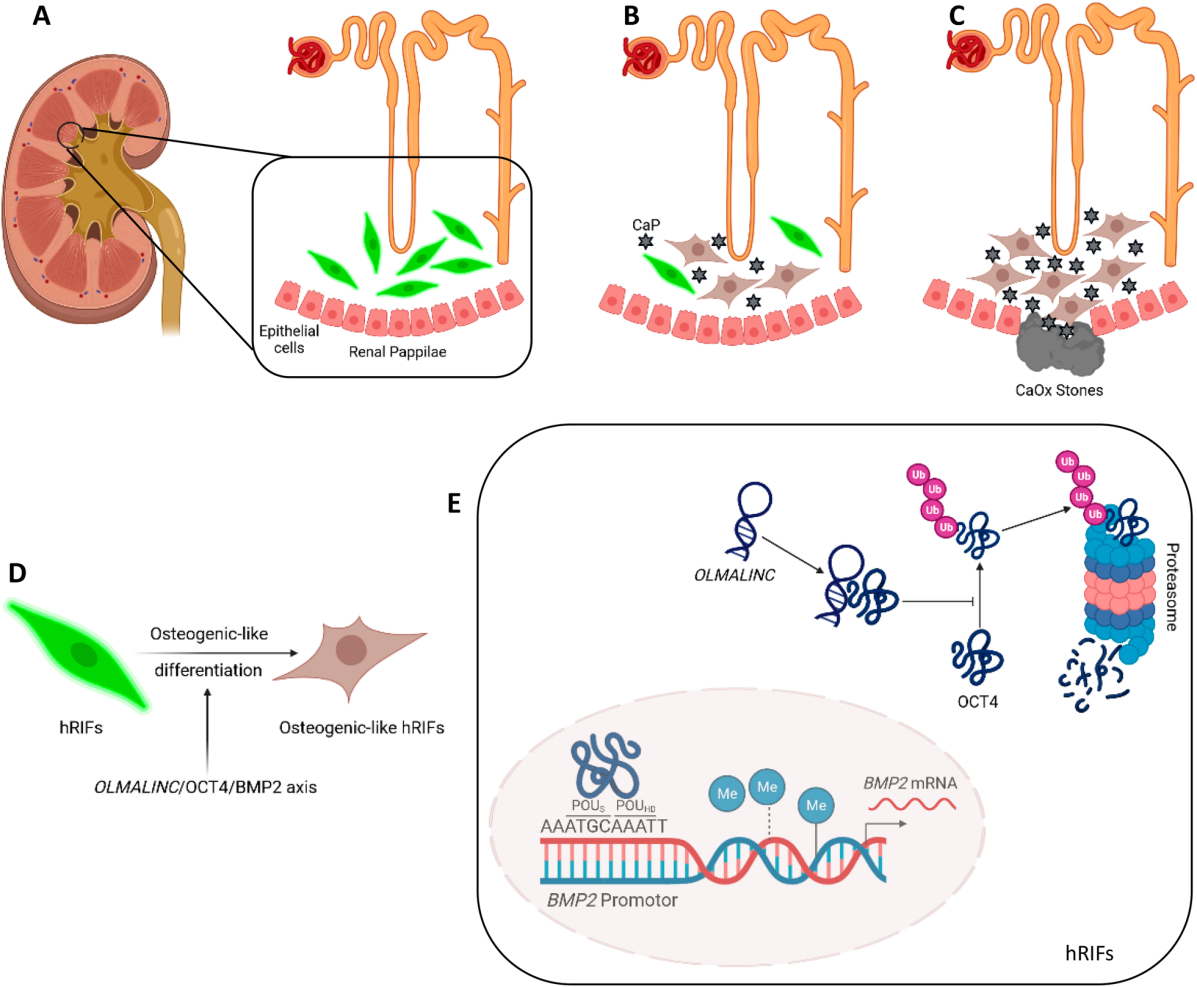
Schematic illustration of the possible involvement of *OLMALINC*/OCT4/*BMP2* axis mediated osteogenic-like differentiation of human renal interstitial fibroblasts (hRIFs) in Randall’s Plaque formation.** A** A normal renal papilla in which Henle loop, hRIFs, and epithelial cells are shown. **B** HRIFs adopt an osteogenic-like phenotype and calcium phosphate (CaP) deposits are formed in renal interstitium of renal papilla. **C** CaP deposits aggregate and impair the musca of renal papilla, leading to RP formation as an anchor for calcium oxalate (CaOx) crystals to induce stone formation. **D, E** The molecular mechanism in the osteogenic-like differentiation of hRIFs based our findings. OCT4 bound to the sequences (AAATGCAAATT) in *BMP2* promoter where the POU-specific domain (POU_S_) and the POU homeodomain (POU_HD_) recognized and bound to ATGC and AAAT respectively, and decreased the DNA methylation of BMP2 promoter to transcriptionally promote *BMP2* expression. *OLMALINC* stabilized OCT4 to enhance osteogenic-like differentiation of hRIFs via disrupting its ubiquitination. This illustration was created with BioRender.com

Taken together, Fig. 7 illustrated the regulatory network of *OLMALINC*/OCT4/*BMP2* axis in the osteogenic-like differentiation of hRIFs, which partially revealed the potential mechanism of renal interstitium biomineralization prior to RP formation.

## Discussion

Increasing evidence suggest that RP formation is a multi-step process of CaP deposits mixed with an organic matrix in which osteogenic-like cells play a substantial role (Khan and Gambaro 2007; Khan et al. 2021). RP not only presented similarities to physiological and pathological biominerals identified by nanoscale analysis (Gay et al. 2020; Evan et al. 2005a), but also conferred upregulated osteogenic-related genes compared to that of NRP (Zhu et al. 2020a; Evan et al. 2005b). Moreover, cells isolated from medullary sponge kidney, a rare congenital malformation characterized by nephrocalcinosis and recurrent renal stones, resembled pericytes or fibroblasts morphologically. These cells adopted osteogenic-like fate and spontaneously formed CaP crystals in cell layers after 4–5 months in culture without osteogenic induction (Mezzabotta et al. 2015). Additionally, our recent study corroborated the evidence that CaP deposits formed in hRIFs as well as HK-2 induced with osteogenic medium (Zhu et al. 2021). Though a significant advance has been made in understanding the structure of RP as well as identifying cells with the potential capacity of osteogenic-like differentiation, a critical question has remained unanswered that the mechanism drives the osteogenic-like process prior to RP formation.

Given that human fibroblasts can be induced to functional osteoblasts by transducing *OCT4* and osteogenic specific factors (Yamamoto et al. 2015); OCT4 functioned as a substantial regulator in maintaining cell pluripotency and cell reprogramming (Jerabek et al. 2014; Kim et al. 2021), and OCT4 was reported to be a promotive regulator in osteogenic differentiation of MSCs as well as osteogenic trans-differentiation of human umbilical vein endothelial cells (Malvicini et al. 2019; Kim et al. 2020), we asked a question whether OCT4 was altered in RP. Intriguingly, we found that OCT4 was upregulated in RP where fibroblasts showed upregulated expression of OCT4 and osteogenic markers as revealed by immunofluorescence co-staining, and OCT4 was further found to be increased in osteogenic induced hRIFs with a time course. Moreover, gain- and loss-of-function verified that OCT4 could significantly enhance osteogenic-like differentiation of hRIFs in vitro and vivo. The above results suggested that OCT4 might regulate osteogenic-like differentiation of hRIFs to participate in RP formation. Of note, OCT4 together with SOX2 and NANOG were reported to reprogram the cancer cells to cancer stem cells (CSCs) and serve as major transcription factors maintaining the stemness of CSCs (Bayik and Lathia 2021; Villodre et al. 2016), and calcification or/and osteogenesis were noted in various cancers (Cook et al. 2019; Wen et al. 2018; O'Grady and Morgan 2018). Additionally, OCT4 was found to be upregulated and accelerate renal cell regeneration in mouse kidney with ischemia reperfusion injury (Rogers et al. 2016). Therefore, further studies are needed to clarify whether there is a causal relationship of upregulated OCT4 and osteogenic markers in RP.

The functions of OCT4 largely depended on its ability to recognize and bind to the regulatory regions of DNA (Kim et al. 2021). The current study firstly determined that OCT4 could bind to the region of *BMP2* promoter (AAATGCAAATT) in hRIFs, and it was echoed to the region conservatively recognized and bound by two subdomains of the POU family, which were the POU-specific domain (POU_S_) and the POU homeodomain (POU_HD_) (Niwa et al. 2008), especially binding to ATGC and AAAT/TAAT (Niwa et al. 2008), respectively. Similarly, our ChIP result indicated OCT4 also bound to the site in *SMAD4* promoter region (TTATGCAAATG) in which the sequences ATGCAAAT were also identified, and thus further investigations were expected to verify this binding and determine whether OCT4 simultaneously promoted the transcription of *BMP2* and *SMAD4* to synergistically enhance osteogenic-like differentiation of hRIFs. Additionally, OCT4 was found to recognize and bind to the enhancer of *OPN*, a key regulator in ectopic calcification, in which there was a palindromic-octamer-recognition element (ATTTGAAATGCAAAT) (Botquin et al. 1998), and thus it is profound to investigate the role of OCT4 in regulating the OPN expression of hRIFs, since OPN was detected in crystalline deposits and was upregulated in RP tissues (Zhu et al. 2020a; Evan et al. 2005b). On the other hand, it has been found that OCT4, NANOG and SOX2 simultaneously bound to hundreds of genes in undifferentiated human embryonic stem cells (Pan and Thomson 2007), including their own promotors, which formed a regulatory network to support or limit each other's expression level (Malik et al. 2018; Pan and Thomson 2007). The interconnected autoregulation loop contributed to maintaining the properties of embryonic stem cells (Malik et al. 2018; Jerabek et al. 2014; Pan and Thomson 2007). It encouraged us to further explore whether OCT4 together with NANOG and SOX2 bind to osteogenesis regulated genes in regulating osteogenic-like differentiation of hRIFs. Additionally, these key factors were involved in multiple signaling pathway to regulate pluripotency of embryonic or/and cancer stem cells, such as Wnt, JAK-STAT, TGF/SMAD NF-κB, PI3K/AKT/mTOR (Do and Schöler 2009; Yang et al. 2020). Thereinto, intriguingly, Wnt signaling pathway was revealed by our previous studies to play an important role in promoting osteogenic-like differentiation of hRIFs (Zhu et al. 2021, 2020b). These findings guide us to further clarify the signaling pathway through which OCT4 regulates the osteogenic-like differentiation of hRIFs.

OCT4 has been identified to transcriptionally regulate the target gene via altering the DNA-methylation level as well as remodeling chromatin (Malik et al. 2018). The current study revealed that OCT4 reduced the DNA methylation level of *BMP2* promoter to enhance its transcription. Consistently, a previous study performed a comprehensive roadmap of Oct4 binding during somatic cell reprogramming, and revealed an inverse correlation between DNA methylation level and Oct4 binding (Chen et al. 2016). Intriguingly, a previous study reported that Oct4 bound to the conserved non-coding sequence regions in *Tet1* and *Tet2* genes, and knockdown of *Oct4* and *Sox2* led to a marked suppression of *Tet1* and *Tet2* mRNA (Koh et al. 2011). Another study revealed an elevated DNA demethylation in aged human adipose-derived MSCs with a decline in proliferation and osteogenic potential, which was inversely correlated with the downregulation of OCT4 as well as TET family, and treatment with 5-Azacytidine could rejuvenate Ad-MSCs partially via improving OCT4, TET2 and TET3 expression (Yan et al. 2014). The above studies inspired us to further verify the speculation that the OCT4 might decrease DNA methylation level of *BMP2* partially via elevating *TET* family which could convert 5-methylcytosine (5-mC) to 5-hydroxymethylcytosine (5-hmC) in DNA. Vascular calcification is also considered as biomineralization where vascular smooth muscle cells undergo osteogenic reprogramming (Opdebeeck et al. 2019). Hyperphosphatemia was identified to convert the contractile phenotype of vascular smooth muscle cells to the osteogenic phenotype, during which DNA 6 mA demethylated by ALKBH1 facilitated OCT4 to recognize and bind to *BMP2* promoter (Ouyang et al. 2021). It shed new light on our further investigations due to the analogous pathological environment identified in renal papilla with high concentration of Ca^2+^ (Evan et al. 2003b; Sepe et al. 2006).

Post-translational modifications have been well-established to regulate the stability as well as diversify the functions of proteins (Duan and Walther 2015), and lncRNAs were reported to mediate multiple post-translational modifications of proteins (Yao et al. 2019). By a combination of RIP and RNA profiling, the current study uncovered that *OLMALINC* not only was upregulated in osteogenic induced hRIFs, but also directly bound to OCT4. Interestingly, *OLMALINC* was further found to disrupt the ubiquitination of OCT4 and thus promoted OCT4 stabilization. In a similar manner, nickel and cobalt treatment inhibited ubiquitination of OCT4 to enhance the stabilization in stem cells (Yao et al. 2014), and disruption of OCT4 ubiquitination potently facilitated pluripotency induction (Rhie et al. xxxx). In addition to the declined stabilization of OCT4, ubiquitination was found to markedly impair the transcriptional activity (Yao et al. 2014). Therefore, theoretically, the promotive role of *OLMALINC* in osteogenic-like differentiation might largely depend on its function in disrupting the ubiquitination of OCT4, but it needed further investigation. Of note, in addition to ubiquitination, post-translational modifications of OCT4 were reported to include phosphorylation, SUMOylation, and glycosylation, which significantly influenced the function and localization of OCT4 (Mehravar et al. 2021). Thus further studies are expected to clarify the potential mechanism of OCT4 in osteogenic-like differentiation of hRIFs.

The current study firstly detected the upregulated OCT4 in RP tissues where at least fibroblasts had increased expression of OCT4, and further identified the promotive role of OCT4 in osteogenic-like differentiation of hRIFs in *vitro* and *vivo*, through which it opened up a new avenue to understand the reprogramming related factor OCT4 in the renal interstitium biomineralization prior to RP formation. Nevertheless, given a consensus view that RP formation involves multiple processes driven by multiple cells, we have to acknowledge a limitation that our study only evaluated the OCT4 expression of fibroblasts in RP, and thus it is required to further evaluate whether OCT4 is altered in other cells, such as renal tubular epithelial cells and vascular endothelial cells. Additionally, further studies are needed to clarify which cell plays a leading role in triggering CaP deposition in renal interstitium. Moreover, the progress in RP studies was largely slowed down by the fact that no ideal animal model has been established to mimic the process of human RP formation. Therefore, we have to realize that effective therapies to prevent the recurrence of CaOx stones will not develop until these related questions are well answered.

## Conclusion

OCT4 was identified to be upregulated and positively correlated with osteogenic markers in RP where fibroblasts partially showed an upregulated OCT4 expression. Further investigation revealed that OCT4 had a promotive effect on the osteogenic-like differentiation of hRIFs both in *vitro* and in *vivo*. Mechanically, OCT4 bound to the *BMP2* promoter and altered its CpG island methylation to transcriptionally promote *BMP2* expression, and *OLMALINC* stabilized OCT4 to enhance osteogenic-like differentiation of hRIFs via disrupting its ubiquitination. Our findings could provide new clues to better clarify the osteogenic-like process prior to RP formation directed by lncRNAs as well as reprogramming related factors.

## Supplementary Information

**Additional file 1: Fig. S1. (A)**. The immunohistochemistry (IHC) staining intensity of OCT4 and osteogenic markers (RUNX2, OCN) was quantified by average grey value using Image J. The average grey value ranged from 0 to 254. A black, dark-stained area was set as a grey value of 0, and a white, unstained area was set as a grey value of 254, causing an inverse correlation of the average gray value with staining intensity (Randall’s Plaques (RP), n = 8; normal renal papillae (NRP), n = 8). **(B)** Representative immunofluorescence co-staining images of Vimentin (Green) and RUNX2 (Red) in RP (n = 6) and NRP (n = 6). **(C)** Representative immunofluorescence co-staining images of Vimentin (Green) and OCN (Red) in RP (n = 6) and NRP (n = 6). **(D)** The Vimentin (VIM) co-localized with OCT4 or osteogenic markers (RUNX2, OCN) was quantified by calculating the ratio of co-localized VIM to total VIM using the confocal microscope system (RP = 6; NRP = 6).

**Additional file 2: Fig. S2. (A)** Quantitative assay of OCT4 protein level determined by Immunoblotting (IB) in human renal interstitial fibroblasts (hRIFs) 0, 3, 7, 14 days after induction with osteogenic medium (OM; n = 3). **(B)** The blue-stained collagen fibers in Masson's trichrome staining of subcutaneous implantations were measured as the collagen volume fraction using Image J. The stained area in porous bone mineral substitute granules, functioned as a carrier of cells, was excluded for analysis. NC = granules with normal control hRIFs; the Len-ctrl group = granules with hRIFs transfected with Len-ctrl; the Len-*OCT4* group = granules with hRIFs transfected with Len-*OCT4*; Len-sh-ctrl group = granules with hRIFs transfected with Len-sh-ctrl; the Len-sh-*OCT4* group = granules with hRIFs transfected with Len-sh-OCT4; n = 6 for each group. **(C)** The immunohistochemistry (IHC) staining intensity (OCT4; RUNX2; OCN) of subcutaneous implantations was quantified by average grey value using Image J. The stained area in porous bone mineral substitute granules was excluded for analysis; n = 6 for each group. **(D-E)** Quantitative assay of BMP2 protein level determined by IB in hRIFs with silenced or overexpressed *OCT4* 7 days after osteogenic induction (n = 3). **(F)** The BMP2 IHC staining intensity of subcutaneous implantations was quantified by average grey value using Image J. The stained area in porous bone mineral substitute granules was excluded for analysis; n = 6. **(G)** QRT-PCR analysis of *BMP2* in hRIFs transfected with multiple Len-sh-*BMP2* (n = 3), and Len-sh3-*BMP2* was used in the following experiments. **(H)** HRIFs were co-transfected with either Len-ctrl or Len-*OCT4* in conjunction with either Len-sh-ctrl or Len-sh-*BMP2*, and alkaline phosphatase (ALP) activity in cell lysis was determined 7 days after osteogenic induction (n = 3). **(I)** HRIFs were transfected with recombinant lentivirus to overexpress or silence *OLMALINC*, and qRT-PCR determined the efficiency (n = 3). Len-*OLMALINC* and Len-sh1-*OLMALINC* were selected for the following experiments. **(J)** Quantitative assay of OCT4 protein level determined by IB in hRIFs transfected with Len-*OLMALINC* or Len-sh-*OLMALINC* (n = 3). **(K)** IB determined OCT4 and ubiquitinated OCT4 in immune-precipitates, and the ratio of ubiquitinated OCT4 to OCT4 was compared between Len-sh-ctrl (n = 4) and Len-sh-*OLMALINC* group (n = 4). **(L)** Quantitative assay of OCN, BMP2, and RUNX2 protein levels determined by IB in hRIFs transfected with Len-*OLMALINC* or Len-sh-*OLMALINC* (n = 3). **(M)** The Vimentin (VIM) co-localized with *OLMALINC* was quantified by calculating the ratio of co-localized VIM to total VIM using the confocal microscope system (Randall’s Plaques (RP), n = 6; normal renal papillae (NRP), n = 6).

**Additional file 3: Fig. S3.** Human renal interstitial fibroblasts (hRIFs) transfected with Len-sh-ctrl or Len-sh-*OCT4* were induced with osteogenic medium for 7 days, and bisulfite sequencing PCR (BSP) determined the sequences in predicted CpG island 2 of *BMP2* promoter. The sequences of 5 randomly picked clones were shown.

**Additional file 4: Fig. S4.** Human renal interstitial fibroblasts (hRIFs) transfected with Len-sh-ctrl or Len-sh-*OCT4* were induced with osteogenic medium for 7 days, and bisulfite sequencing PCR (BSP) determined the sequences in predicted CpG island 1 of *BMP2* promoter. The sequences of 5 randomly picked clones were shown.

**Additional file 5: Table S1.** The characteristics of patients with CaOx stones and patients with renal cancers. **Table S2.** ShRNA sequences designed for silencing *OCT4* *BMP2 *and* OLMALINC*. **Table** **S3** Primer sequences designed for qRT-PCR. **Table** **S4**. Primer sequences designed for 5’ and 3’ RACE of *OLMALINC*. **Table S5.** The details of primary antibodies used in immunoblotting (IB). **Table S6.** Primer sequences designed for CHIP-qPCR. **Table S7. **Primer sequences designed for bisulfite sequencing PCR (BSP) of *BMP2* promoter. **Table S8. **The predicted sites in *BMP2* or *SMAD4* promoter which OCT4 binds to. **Table S9. **The intersection of OCT4 binding lncRNAs identified by RIP-Seq and upregulated lncRNAs identified by RNA profiling. **Table S10. **The full length of *OLMALINC* identified by 5’ and 3’ RACE in the current study and the transcript variant deposited in the NCBI database.

## Data Availability

The datasets of RNA-seq generated during the current study are available in the GEO DataSets (GSE203110), and other datasets used and/or analyzed during the current study are available from the corresponding author on reasonable request.
