# Supplementary figures and images for: OLMALINC/OCT4/BMP2 axis enhances osteogenic-like phenotype of renal interstitial fibroblasts to participate in Randall’s plaque formation
Source: Mol Med. 2022 Dec 29;28:162. doi: 10.1186/s10020-022-00576-4 (PMC9798568; doi:10.1186/s10020-022-00576-4)

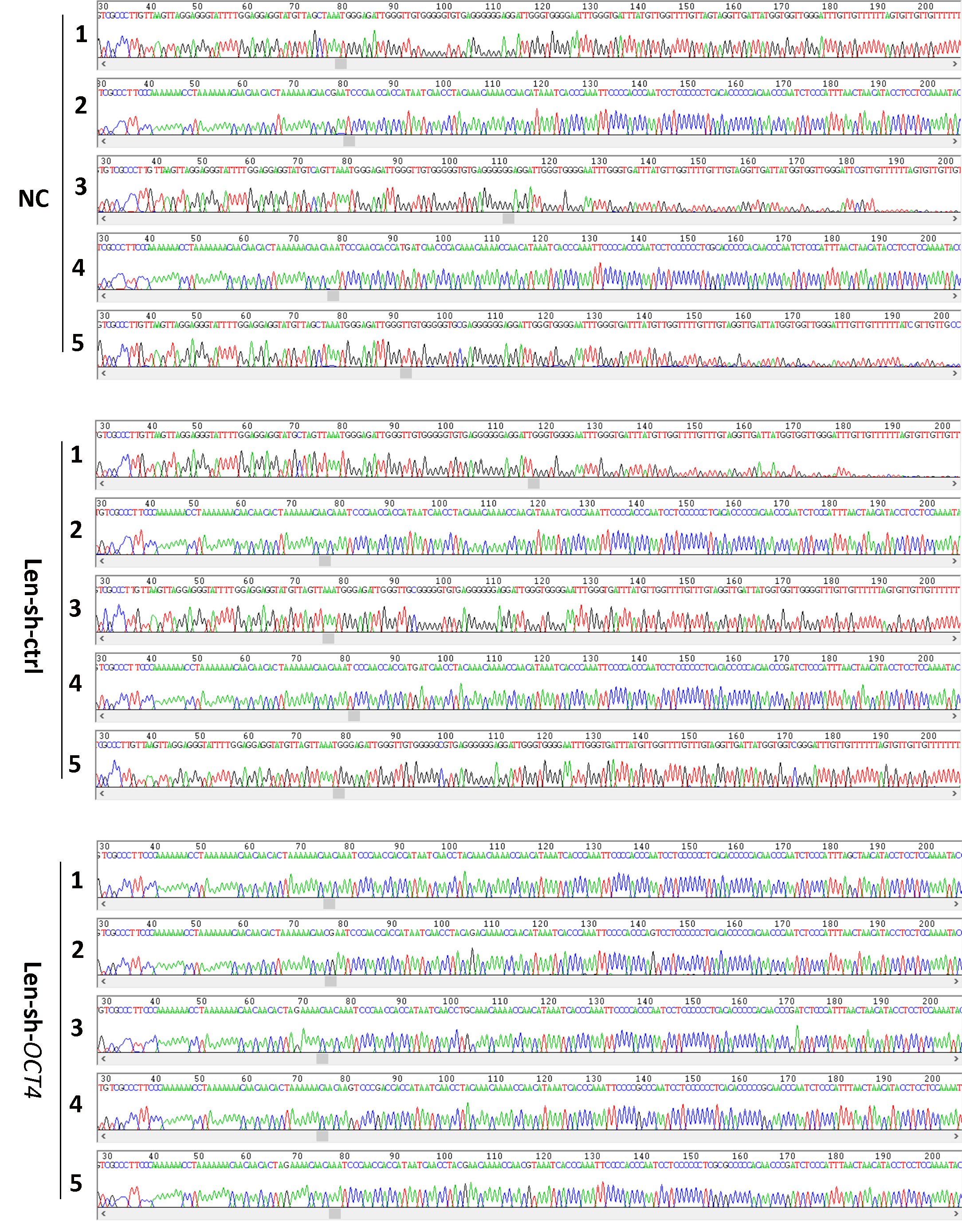

Supplement: Supplementary file 3 — Additional file 3: Fig. S3. Human renal interstitial fibroblasts (hRIFs) transfected with Len-sh-ctrl or Len-sh-OCT4 were induced with osteogenic medium for 7 days, and bisulfite sequencing PCR (BSP) determined the sequences in predicted CpG island 2 of BMP2 promoter. The sequences of 5 randomly picked clones were shown. [file 10020_2022_576_MOESM3_ESM.tif]

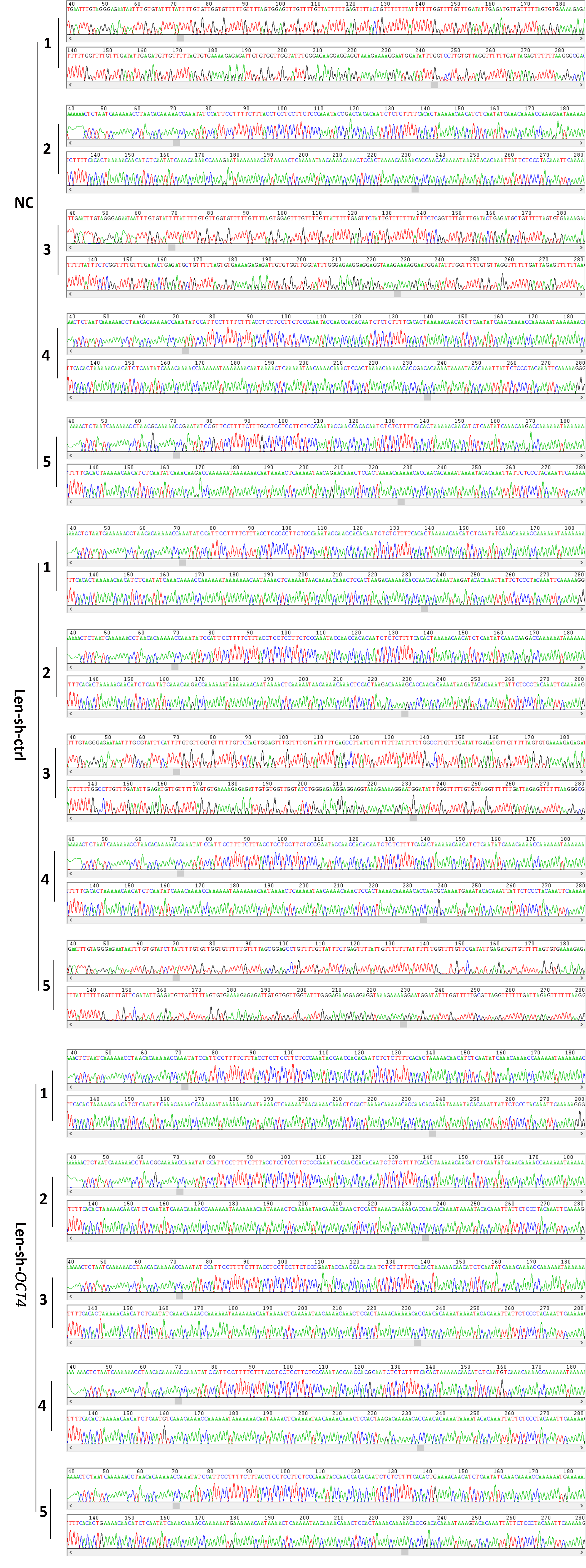

Supplement: Supplementary file 4 — Additional file 4: Fig. S4. Human renal interstitial fibroblasts (hRIFs) transfected with Len-sh-ctrl or Len-sh-OCT4 were induced with osteogenic medium for 7 days, and bisulfite sequencing PCR (BSP) determined the sequences in predicted CpG island 1 of BMP2 promoter. The sequences of 5 randomly picked clones were shown. [file 10020_2022_576_MOESM4_ESM.tif]
