# Supplementary material for: OLMALINC/OCT4/BMP2 axis enhances osteogenic-like phenotype of renal interstitial fibroblasts to participate in Randall’s plaque formation
Source: Mol Med. 2022 Dec 29;28:162. doi: 10.1186/s10020-022-00576-4 (PMC9798568; doi:10.1186/s10020-022-00576-4)
Supplement: Supplementary file 5 — Additional file 5: Table S1. The characteristics of patients with CaOx stones and patients with renal cancers. Table S2. ShRNA sequences designed for silencing OCT4 BMP2 and OLMALINC. Table S3 Primer sequences designed for qRT-PCR. Table S4. Primer sequences designed for 5’ and 3’ RACE of OLMALINC. Table S5. The details of primary antibodies used in immunoblotting (IB). Table S6. Primer sequences designed for CHIP-qPCR. Table S7. Primer sequences designed for bisulfite sequencing PCR (BSP) of BMP2 promoter. Table S8. The predicted sites in BMP2 or SMAD4 promoter which OCT4 binds to. Table S9. The intersection of OCT4 binding lncRNAs identified by RIP-Seq and upregulated lncRNAs identified by RNA profiling. Table S10. The full length of OLMALINC identified by 5’ and 3’ RACE in the current study and the transcript variant deposited in the NCBI database. [file 10020_2022_576_MOESM5_ESM.docx]

**Supplementary Table 1.** The characteristics of patients with CaOx stones and patients with renal cancers.

| **characteristics** | **Patients with CaOx stones (n=28)** | **Patients with renal cancers (n=28)** | **P value** |
| --- | --- | --- | --- |
| Age (years), mean±SD | 54.4±10.5 | 59.1±7.5 | 0.071 |
| Gender (M/F) | 17/11 | 15/13 | 0.589 |
| Hydronephrosis degree*, n, % |  |  | 0.365 |
| None or mild | 19(67.9%) | 22(78.6%) |  |
| moderate | 9(32.1%) | 6(21.4%) |  |
| Comorbidities, n (%) |  |  | 0.860 |
| Renal insufficiency | 3(10.7%) | 2(7.1%) |  |
| Hypertension | 9(32.1%) | 11(39.3%) |  |
| Diabetes mellitus | 2(7.1%) | 3(10.7%) |  |
| Stone burden (mm^2^)**, mean±SD | 856±313 | - | - |
| Tumor size (cm), mean±SD | - | 5.8±1.2 | - |

* The Society of Fetal Urology grading system was used to determine the hydronephrosis degree, and those with severe hydronephrosis were routinely excluded in current study. **The formula (0.785×length_max_×width_max_) was used to calculated the stone burden according to CROES. CaOx, calcium oxalate;M, male; F, female; SD, standard deviation.

**Supplementary Table 2.** ShRNA sequences designed for silencing *OCT4* *BMP2* and *OLMALINC.*

| **Gene** |  | **shRNA sequences** |
| --- | --- | --- |
| *OCT4* | Len-sh1-*OCT4* | Sense: 5’-3’ CCGGAACATGTGTAAGCTGCGGCCCCTCGAGGGGCCGCAGCTTACACATGTTTTTTTG |
|  |  | Anti-sense: 5’-3’ AATTCAAAAAAACATGTGTAAGCTGCGGCCCCTCGAGGGGCCGCAGCTTACACATGTT |
|  | Len-sh2- *OCT4* | Sense: 5’-3’ CCGGTGGTCCGAGTGTGGTTCTGTACTCGAGTACAGAACCACACTCGGACCATTTTTG |
|  |  | Anti-sense: 5’-3’ AATTCAAAAATGGTCCGAGTGTGGTTCTGTACTCGAGCATAGTCGCTGCTTGATCGCT |
|  | Len-sh3- *OCT4* | Sense: 5’-3’ CCGGAGCGATCAAGCAGCGACTATGCTCGAGCATAGTCGCTGCTTGATCGCTTTTTTG |
|  |  | Anti-sense: 5’-3’ AATTCAAAAAAGCGATCAAGCAGCGACTATGCTCGAGCATAGTCGCTGCTTGATCGCT |
| *BMP2* | Len-sh1- *BMP2* | Sense: 5’-3’ CCGGTCCAAGAGACATGTTAGGATACTCGAGTATCCTAACATGTCTCTTGGATTTTTG |
|  |  | Anti-sense: 5’-3’ AATTCAAAAATCCAAGAGACATGTTAGGATACTCGAGTATCCTAACATGTCTCTTGGA |
|  | Len-sh2- *BMP2* | Sense: 5’-3’ CCGGAAGGCCATTGCTAGTAACTTTCTCGAGAAAGTTACTAGCAATGGCCTTTTTTTG |
|  |  | Anti-sense: 5’-3’ AATTCAAAAAAAGGCCATTGCTAGTAACTTTCTCGAGAAAGTTACTAGCAATGGCCTT |
|  | Len-sh3- *BMP2* | Sense: 5’-3’ CCGGAAACGTCAAGCCAAACACAAACTCGAGTTTGTGTTTGGCTTGACGTTTTTTTTG |
|  |  | Anti-sense: 5’-3’ AATTCAAAAAAAACGTCAAGCCAAACACAAACTCGAGTTTGTGTTTGGCTTGACGTTT |
| *OLMALINC* | Len-sh1- *OLMALINC* | Sense: 5’-3’ CCGGCCCGAGATTCTTTGTGGGCTCTCGAGAGCCCACAAAGAATCTCGGGTTTTTG |
|  |  | Anti-sense: 5’-3’ AATTCAAAAACCCGAGATTCTTTGTGGGCTCTCGAGAGCCCACAAAGAATCTCGGG |
|  | Len-sh2- *OLMALINC* | Sense: 5’-3’ CCGGCTGGAATGGGGAAAATGCGGCTCGAGCCGCATTTTCCCCATTCCAGTTTTTG |
|  |  | Anti-sense: 5’-3’ AATTCAAAAACTGGAATGGGGAAAATGCGGCTCGAGCCGCATTTTCCCCATTCCAG |
|  | Len-sh3- *OLMALINC* | Sense: 5’-3’ CCGGGACCTTGCTAACCAGGACGGCTCGAGCCGTCCTGGTTAGCAAGGTCTTTTTG |
|  |  | Anti-sense: 5’-3’ AATTCAAAAAGACCTTGCTAACCAGGACGGCTCGAGCCGTCCTGGTTAGCAAGGTC |
| Len-sh-ctrl | Involved in the kit | |

**Supplementary Table** 3. Primer sequences designed for qRT-PCR.

| **Gene** | **Forward primer 5′–3′** | **Reverse primer 5′–3′** |
| --- | --- | --- |
| *GAPDH* | AACGTGTCAGTGGTGGACCTG | AGTGGGTGTCGCTGTTGAAGT |
| *OCT4* | TGTCAGGGCTCTTTGTCCAC | TCTCCCCAGCTTGCTTTGAG |
| *RUNX2* | CACCATGTCAGCAAAACTTCTT | TCACGTCGCTCATTTTGC |
| *BMP2* | GTGGACAAGACTCGGGATGAAA | ATCACGTAATGCCTGCTGTG |
| *OCN* | CTTTGTGTCCAAGCAGGA | CTGAAAGCCGATGTGGTCAG |
| *TARID* | CAGAGTCACAGAAGATGGCACAGC | GCAGTAGAGGAAGTTGGTCGGATG |
| *OLMALINC* | TCAGTCTGCCCTACCCTGGAATG | CTCTGAGTTCTGGAGCACTGTTCG |
| *MIR17HG* | GGGGCCTCCGGTCGTAGTAAAG | AGGAGTAGCCGCCACCATCTTC |
| *ZBED3-AS1* | GGGAGAGCAGGAGAGTGGTGAC | GCCGTCTTCCAGTGCCTCATTC |
| *LOC100130872* | TCCTGCCAGCTCCGGTCTTG | TCACGCCTCTTCTCTAGCCTTCC |

**Supplementary Table** 4. Primer sequences designed for 5’ and 3’ RACE of *OLMALINC.*

|  | **Gene specific primer (GSP) 5′–3′** | **Nest Gene specific primer (NGSP) 5′–3′** |
| --- | --- | --- |
| 5’-RACE | GATTACGCCAAGCTTTTGTGGATCTTCAGTTGCTTCAGGTCATCT | GATTACGCCAAGCTTCGTGTGAAGAAACCACCAAACAGGCTTT |
| 3’-RACE | GATTACGCCAAGCTTGCGAACAGTGCTCCAGAACTCAGAGA | GATTACGCCAAGCTTCCCTCCCTCATAACCCGAGATTCTTTGTG |

**Supplementary Table 5.** The details of primary antibodies used in immunoblotting (IB).

| **Antibody** | **Dilution ratio** | **Company (Cat No.)** |
| --- | --- | --- |
| GAPDH | 1:5000 | Proteintech, China (60004-1-Ig) |
| OCT4 | 1:4000 | Abcam, UK (ab184665) |
| OCN | 1:3000 | Abcam, UK (ab133612) |
| RUNX2 | 1:3000 | CST, USA (#12556) |
| BMP2 | 1:2000 | Servicebio, China (18933-1-AP) |
| ubiquitin | 1:3000 | Santa Cruz Biotechnology, UAS (sc-8017) |
| HRP conjugated goat anti-rabbit IgG | 1:5000 | Proteintech, China (SA00001-2) |
| HRP conjugated goat anti-mouse IgG | 1:5000 | Proteintech, China (SA00001-1) |

**Supplementary Table 6.** Primer sequences designed for CHIP-qPCR

| **EGR1-bound sites (E-BS)** | **Forward primer 5′–3′** | **Reverse primer 5′–3′** |
| --- | --- | --- |
| *BMP2*-PBS1 (202bp) | \| GTGGACAAGACTCGGGATGAAA \| \| --- \| | ATCACGTAATGCCTGCTGTG |
| *BMP2*-PBS2 (391bp) | AATTCTCATAGCCTCGGCTCC | CCGGGGTTCCCATGAAAGAAT |
| *BMP2*-PBS3 (338bp) | TACCCGTAAACATGTTGCATTTCT | AGGATGAGTGGAGTGGGGTAA |
| *BMP2*-PBS4 (248bp) | CCGAGTCTTGTCCACACACAA | ACTTAGTGCATCTGCCAGCAA |
| *SMAD4*-PBS1 (83bp) | \| CAAATCACAGGCGCCACCTA \| \| --- \| | AGGCCCCATATGATTCCCAC |
| Positive control (For *GAPDH*; 110bp) | Involved in the kit | Involved in the kit |

**Supplementary Table 7.** Primer sequences designed for bisulfite sequencing PCR (BSP) of *BMP2* promoter

|  | **Forward primer 5′–3′** | **Reverse primer 5′–3′** |
| --- | --- | --- |
| CpG island 1 | GTTAAGTTAGGAGGGTATTTTGGAG | CCCAAAAAAACCTAAAAAAACAAC |
| CpG island 2 | TTTGAATTTGTAGGGAGAATAATTTG | AAAAAACTCTAATCAAAAAACCTAAC |

**Supplementary Table 8.** The predicted sites in *BMP2* or *SMAD4* promoter which OCT4 binds to.

| **Transcript** | **gene** | **Strand** | **Score** | **P-Value** | **Q-Value** | **Matched Sequence** | **Motif Site** | **Motif_Web** |
| --- | --- | --- | --- | --- | --- | --- | --- | --- |
| NM_001200.3 | BMP2 | - | 13.848 | 4.63*10^-6^ | 0.376 | TTATGCAAATC(*BMP2*-PBS1) | TSS -4105 bp | http://jaspar.genereg.net/matrix/MA1115.1 |
| NM_001200.3 | BMP2 | - | 13.664 | 6.35*10^-6^ | 0.376 | ACATGCAAATT (*BMP2*-PBS2) | TSSS -2774 bp | http://jaspar.genereg.net/matrix/MA1115.1 |
| NM_001200.3 | BMP2 | + | 13.384 | 8.95*10^-6^ | 0.396 | GTATGCAAATG (*BMP2*-PBS3) | TSS -4548 bp | http://jaspar.genereg.net/matrix/MA1115.1 |
| NM_001200.3 | BMP2 | + | 13.752 | 5.08*10^-6^ | 0.376 | AAATGCAAATT (*BMP2*-PBS4) | TSS -2620 bp | http://jaspar.genereg.net/matrix/MA1115.1 |
| NM_005359.5 | SMAD4 | - | 14.384 | 7.4*10^-7^ | 0.386 | TTATGCAAATG (*SMAD4*-PBS1) | TSS -2882 bp | http://jaspar.genereg.net/matrix/MA1115.1 |

*BMP2*-PBS1/2/3/4=*BMP2* promoter binding site 1/2/3/4; *SMAD4*-PBS1=*SMAD4* promoter binding site 1. TSS -= upstream of transcriptional start site.

**Supplementary Table 9.** The intersection of OCT4 binding lncRNAs identified by RIP-Seq and upregulated lncRNAs identified by RNA profiling.

| **LncRNA** | **Gene** | **log2(Fold_enrichment in RIP)** | **log2(Fold_change in RNA profiling)** | **Website** |
| --- | --- | --- | --- | --- |
| NR_109982.1 | *TARID* | 5.2161 | 1.7516 | http://www.ncbi.nlm.nih.gov/nuccore/NR_109982.1 |
| NR_026762.1 | *OLMALINC* | 4.2196 | 1.1250 | http://www.ncbi.nlm.nih.gov/nuccore/NR_026762.1 |
| NR_027350.1 | *MIR17HG* | 3.9682 | 1.8191 | http://www.ncbi.nlm.nih.gov/nuccore/NR_027350.1 |
| NR_024398.1 | *ZBED3-AS1* | 3.8463 | 1.5268 | http://www.ncbi.nlm.nih.gov/nuccore/NR_024398.1 |
| NR_024569.1 | *LOC100130872* | 3.5301 | 1.6859 | http://www.ncbi.nlm.nih.gov/nuccore/NR_024569.1 |
| NR_027107.2 | *LOC90768* | 3.4746 | 1.0800 | http://www.ncbi.nlm.nih.gov/nuccore/NR_027107.2 |
| ENST00000552061.1 | *ENSG00000257298.1* | 3.3961 | 1.0165 | http://asia.ensembl.org/Homo_sapiens/Transcript/Idhistory?t=ENST00000552061 |
| ENST00000607641.1 | *ENSG00000227954.2* | 3.3009 | 1.6088 | http://asia.ensembl.org/Homo_sapiens/Transcript/Idhistory?t=ENST00000607641 |
| ENST00000519801.1 | *ENSG00000247081.3* | 3.2493 | 1.8513 | http://asia.ensembl.org/Homo_sapiens/Transcript/Idhistory?t=ENST00000519801 |
| ENST00000502514.1 | *ENSG00000249684.1* | 3.0964 | 1.9095 | http://asia.ensembl.org/Homo_sapiens/Transcript/Idhistory?t=ENST00000502514 |
| ENST00000606154.1 | *ENSG00000271811.1* | 3.0192 | 1.4636 | http://asia.ensembl.org/Homo_sapiens/Transcript/Idhistory?t=ENST00000606154 |
| ENST00000432120.1 | *ENSG00000244332.1* | 2.9817 | 1.3726 | http://asia.ensembl.org/Homo_sapiens/Transcript/Idhistory?t=ENST00000432120 |
| NR_121187.1 | *PGM5P4-AS1* | 2.9435 | 4.1341 | http://www.ncbi.nlm.nih.gov/nuccore/NR_121187.1 |
| NR_028302.1 | *DUBR* | 2.8494 | 1.1959 | http://www.ncbi.nlm.nih.gov/nuccore/NR_028302.1 |
| NR_002909.2 | *SNHG3* | 2.8288 | 1.1652 | http://www.ncbi.nlm.nih.gov/nuccore/NR_002909.2 |
| NR_038109.1 | *SNHG16* | 2.7817 | 1.1089 | http://www.ncbi.nlm.nih.gov/nuccore/NR_038109.1 |
| NR_038110.1 | *SNHG16* | 2.7469 | 1.1230 | http://www.ncbi.nlm.nih.gov/nuccore/NR_038110.1 |
| NR_110150.1 | *ADAMTS9-AS1* | 2.6472 | 6.4175 | http://www.ncbi.nlm.nih.gov/nuccore/NR_110150.1 |
| NR_073567.1 | *GHRLOS* | 2.5436 | 1.2644 | http://www.ncbi.nlm.nih.gov/nuccore/NR_073567.1 |
| ENST00000434020.1 | *ENSG00000230587.1* | 2.4207 | 3.2262 | http://asia.ensembl.org/Homo_sapiens/Transcript/Idhistory?t=ENST00000434020 |
| NR_111004.2 | *MIR99AHG* | 2.0193 | 2.6353 | http://www.ncbi.nlm.nih.gov/nuccore/NR_111004.2 |

**Supplementary Table 10.** The full length of *OLMALINC* identified by 5’ and 3’ RACE in the current study and the transcript variant deposited in the NCBI database.

| ***OLMALINC*** | **Sequences** |
| --- | --- |
| The current study (1023bp) | CCTTCCTTTTTTTTTTTTTTTCCCGGGGAGTCGGCCTCGGGGCTCTGCTCTCCTACCTCAGTCTGCCCTACCCTGGAATGGGGAAAATGCGGACATGCCACTCAGTCCGGCCGCGAACAGTGCTCCAGAACTCAGAGAGTTTTCCAGACGGGTGGAGATCGCGTTCCCTGCCCGCCGAGGTCCCATCGCTTCCCTGCTGGGAAGACAAATGAGGCGCTTTAGCCGTCTCCACGGCCAGCCCCTCCCTCATAACCCGAGATTCTTTGTGGGCTCTTAGTCCATAGCTGCCTTTGAGGTGGTGTAGACCTTGCTAACCAGGACGGCCCAGTAGGCAGAGCTCATTTTTATTCCTGTCTGCAATCGTGCAAAAACGCCTCTTATGGAAAAGCCAGAGCGCCAGGAGTCAGCAAAACACACTAAAGATTGGGCAGTCACTGGGGAGAACACTCAGCCCGCCTGCACCCAGGTGAAATATACAGCCTTGTTGCTCACACAAAGCCTGTTTGGTGGTTTCTTCACACGGATGCATGTGACATTTGGTGCTGAAGACCCAGGACAGGAGGACTCCTTTGGGAGACCAGTGCCCTGTTGTCGCCCTCACTCCGTGAGGAGATCCACCTATGATCTCAGGTCCTCAGACCAACCAGCCCAAGGAACATCTTGCCAATTTCAAATCGGATAGGAGTGTCAGGCCTCTGAGTCCAAGCTAAGCCATCAAATCCCCTGTGACCTGCACGTGTACATCCAGATGACCTGAAGCAACTGAAGATCCACAAAAGAAGTGAAAGTAGCCTTAACTGATGACATTCCACCATTGTGATTTGTTCCTGCCCCACGCTAACTGATACCATATATTCTTCCCCCGCCCTTGAGAATGTACTTTGTACACCTATCCCAAACCTATAAGAACTAATGATAATCCTACCACCCTTTGCTGACTCTCTTTTTGGACTCAGCCCGCCTGCACCCAGGTGAAATAAACAGCCCTGTTGCTCAAAAAAAAAAAAAAAAAAAAAAAAAAAA |
| NR_026762.1 (1003bp) | TTTTTTTTTTTTTCCCGGGGAGTCGGCCTCGGGGCTCTGCTCTCCTACCTCAGTCTGCCCTACCCTGGAATGGGGAAAATGCGGACATGCCACTCAGTCCGGCCGCGAACAGTGCTCCAGAACTCAGAGAGTTTTCCAGACGGGTGGAGATCGCGTTCCCTGCCCGCCGAGGTCCCATCGCTTCCCTGCTGGGAAGACAAATGAGGCGCTTTAGCCGTCTCCACGGCCAGCCCCTCCCTCATAACCCGAGATTCTTTGTGGGCTCTTAGTCCATAGCTGCCTTTGAGGTGGTGTAGACCTTGCTAACCAGGACGGCCCAGTAGGCAGAGCTCATTTTTATTCCTGTCTGCAATCGTGCAAAAACGCCTCTTATGGAAAAGCCAGAGCGCCAGGAGTCAGCAAAACACACTAAAGATTGGGCAGTCACTGGGGAGAACACTCAGCCCGCCTGCACCCAGGTGAAATATACAGCCTTGTTGCTCACACAAAGCCTGTTTGGTGGTTTCTTCACACGGATGCATGTGACATTTGGTGCTGAAGACCCAGGACAGGAGGACTCCTTTGGGAGACCAGTGCCCTGTTGTCGCCCTCACTCCGTGAGGAGATCCACCTATGATCTCAGGTCCTCAGACCAACCAGCCCAAGGAACATCTTGCCAATTTCAAATCGGATAGGAGTGTCAGGCCTCTGAGTCCAAGCTAAGCCATCAAATCCCCTGTGACCTGCACGTGTACATCCAGATGACCTGAAGCAACTGAAGATCCACAAAAGAAGTGAAAGTAGCCTTAACTGATGACATTCCACCATTGTGATTTGTTCCTGCCCCACGCTAACTGATACCATATATTCTTCCCCCGCCCTTGAGAATGTACTTTGTACACCTATCCCAAACCTATAAGAACTAATGATAATCCTACCACCCTTTGCTGACTCTCTTTTTGGACTCAGCCCGCCTGCACCCAGGTGAAATAAACAGCCCTGTTGCTCAAAAAAAAAAAAAAAA |
